# Metformin and cancer in type 2 diabetes: a systematic review and comprehensive bias evaluation

**DOI:** 10.1093/ije/dyw275

**Published:** 2016-12-12

**Authors:** Ruth E Farmer, Deborah Ford, Harriet J Forbes, Nishi Chaturvedi, Richard Kaplan, Liam Smeeth, Krishnan Bhaskaran

**Affiliations:** 1 Department of Non-communicable Disease Epidemiology, London School of Hygiene and Tropical Medicine, London, UK; 2 MRC Clinical Trials Unit at UCL, London, UK and; 3 Institute of Cardiovascular Science, University College London, London, UK

**Keywords:** Pharmacoepidemiology, diabetes, cancer, confounding, bias, causality

## Abstract

**Background:** Existing observational studies provide conflicting evidence for the causal effect of metformin use on cancer risk in patients with type-2 diabetes, and there are concerns about bias affecting a number of studies.

**Methods:** MEDLINE was used to identify observational studies investigating the association between metformin and overall or site-specific cancer in people with type-2 diabetes. A systematic data extraction and bias assessment was conducted, in which risk of eight bias domains (outcome, exposure, control selection, baseline confounding, time-dependent confounding, immortal time, missing data, censoring methods) were assessed against pre-defined criteria, and rated as unlikely, low, medium or high.

**Results:** Of 46 studies identified, 21 assessed the effect of metformin on all cancer. Reported relative risks ranged from 0.23 to 1.22, with 12/21 reporting a statistically significant protective effect and none a harmful effect. The range of estimates was similar for site-specific cancers; 3/46 studies were rated as low or unlikely risk of bias in all domains. Two of these had results consistent with no effect of metformin; one observed a moderate protective effect overall, but presented further analyses that the authors concluded were inconsistent with causality. However, 28/46 studies were at risk from bias through exposure definition, 22 through insufficient baseline adjustment and 35 from possible time-dependent confounding.

**Conclusions:** Observational studies on metformin and cancer varied in design, and the majority were at risk of a range of biases. The studies least likely to be affected by bias did not support a causal effect of metformin on cancer risk.

## Introduction

Research exists to suggest type 2 diabetes mellitus (T2DM) may be a risk factor for cancer,^1^^,^^2^ and observational studies have suggested that diabetic therapies could also affect this risk.^3–5^ Multiple observational studies have reported an apparent protective effect of metformin, a common first-line therapy for T2DM, against incidence of any cancer.^5–9^ However, a number of potential biases have been identified within some of these studies.^10^ There are limited data from clinical trials comparing metformin with other treatments, though one meta-analysis of adverse events from trials did not find any association between metformin use and cancer occurrence.^11^.

Particular difficulties arise for observational studies in this context because treatment with metformin for T2DM changes through time (is ‘time varying’), and is influenced by disease severity. This means that disease severity may be a confounder between metformin use and cancer, but will also be on the causal pathway since metformin is prescribed in order to control disease severity. For example, glycated haemoglobin (HbA1c), a measure of long-term blood glucose control, and body mass index (BMI) are predictive of metformin use according to well-defined treatment guidelines for T2DM,^12^ but use of metformin will likely influence future HbA1c and BMI. There is also evidence that both BMI^13^ and HbA1c^14^ affect cancer risk. In this situation, standard statistical models cannot estimate the true causal effect of time-varying treatment.^15^ Throughout this paper, such time-updated variables that may be both confounders of, and on the causal pathway for the association between exposure and outcome are referred to as ‘time dependent confounders affected by prior treatment’ (Box 1).

Reviews to date have examined existing evidence for the link between metformin use and cancer; however, some were not comprehensive^10^ and others have not systematically evaluated or presented a detailed evaluation of bias.^16–21^

The aim of this study was to summarize existing observational studies investigating possible associations between metformin use and cancer risk in patients with T2DM, and to systematically examine the research design and analysis methods with regard to risk of bias. A secondary aim was to use meta-regression to estimate the extent to which these biases may account for the differences between study estimates.

BOX 1. Key definition: ‘time-dependent confounder affected by previous treatment

A variable is a time-dependent confounder if it satisfies the following conditions:

- the variable changes through time;
- values are predictive of treatment initiation;
- the variable is also associated with the outcome of interest.

When the time-dependent confounder is also affected by previous treatment, as depicted in the causal diagram below, standard statistical methods cannot provide unbiased estimates of the total causal effect of time-varying treatment.

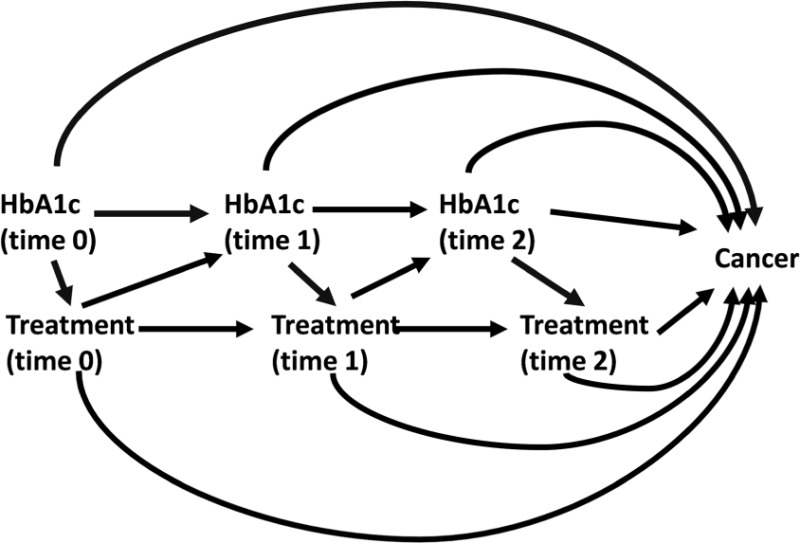

## Methods

### Search strategy

MEDLINE was searched by R.F. using OvidSP on 30 May 2014 for all English-language published articles on cancer risk and type 2 diabetes treatments from 1946 onwards. The search involved using MeSH headings as well as keyword searches in the title and abstract. The full search terms are presented in Supplementary data (available at *IJE* online). Conference abstracts and unpublished studies were excluded.

### Screening strategy

Articles were included in the review if they were of a standard epidemiological design and presented original observational research. Reviews and meta-analysis were not included. Studies were required to present a measure of effect of metformin on risk of cancer incidence (either all cancer or site-specific) in patients with T2DM, with age adjustment as a minimum. Studies restricted to populations with additional comorbidities or diseases were excluded.

During an initial title and abstract screen, reviews, meta analyses and editorial pieces that looked at metformin and cancer were retained so that reference lists could be checked. Additionally, papers that appeared not to meet inclusion criteria, for example those that had primarily compared cancer incidence between diabetics and non-diabetics, were kept for full-text screening in case the required measure of effect was reported as a secondary analysis. A full-text screening was then applied to the remaining papers, and the reference lists of relevant reviews and meta analyses searched. A 10% random sample of the extracted studies were screened by an additional researcher (H.F.) to test the reliability of the inclusion criteria. A Cohens kappa score^22^ was calculated to give a quantitative measure of rater reliability, with a value of 0.75 used as the threshold for ‘excellent agreement’.^23^.

### Data extraction and bias assessment

The data extraction table was piloted on five studies (by R.F., K.B. and D.F.) and subsequently refined to ensure systematic documentation of the relevant information. An example extraction table is supplied in Supplementary Table 1 (available as Supplementary data at *IJE* online).

**Table 1.** Frequency tables to summarize data source, outcome and exposure definitions for 46 studies

	Case-control *N* (%)	Cohort *N* (%)	Total *N* (%)
Data source			
Clinical trial	0 (0)	1 (4)	1 (2)
Diabetes Registry	2 (9)	4 (17)	6 (13)
Insurance database	2 (9)	9 (38)	11 (24)
CPRD (or GPRD)	8 (36)	6 (25)	14 (30)
Other primary/secondary care database	1 (5)	4 (17)	5 (11)
Recruited from hospital/clinic	9 (41)	0 (0)	9 (20)
Outcome definition^a^			
All cancer	5 (23)	16 (67)	21 (46)
Colorectal/bowel	2 (9)	12 (50)	14 (3)
HCC/ICC	5 (23)	2 (8)	7 (15)
Ovarian/endometrial	2 (9)	1 (4)	3 (7)
Bladder	0 (0)	3 (13)	3 (7)
Breast	3 (14)	10 (42)	13 (28)
Oesophagus	0 (0)	4 (17)	4 (9)
Kidney	0 (0)	2 (8)	2 (4)
Liver	0 (0)	5 (21)	5 (11)
Leukaemia	0 (0)	1 (4.2)	1 (2)
Lung	4 (18)	8 (33)	12 (26)
Melanoma	0 (0)	2 (8)	2 (4)
Pancreas	3 (14)	10 (42)	13 (28)
Prostate	3 (14)	8 (33)	11 (24)
Stomach	1 (5)	4 (17)	5 (11)
Definition of exposure to metformin for primary estimate			
Any exposure	14 (64)	8 (33)	22 (48)
Any exposure but minimum time/number of prescriptions needed	1 (5)	2 (8)	3 (7)
Total exposure (number of prescriptions/time on metformin)	6 (27)	4 (17)	10 (22)
Monotherapy	1 (5)	8 (33)	9 (20)
Randomization	0 (0)	1 (4)	1 (2)
Combination therapy with sulphonylurea	0 (0)	1 (4)	1 (2)
Timing of exposure measurement			
Current use (at time of cancer/matched date)	3 (14)	0 (0)	3 (7)
Time updated (current/ever/cumulative)	0 (0)	8 (33)	8 (17)
Fixed from start of follow-up, with exposure occurring in a baseline period or follow-up starting from first exposure ((ITT)	0 (0)	8 (33)	8 (17)
Single summary measure of exposure over entire follow-up	19 (86)	8 (33)	27 (59)
Comparator group for primary estimate			
Less exposure (i.e. continuous exposure variable)	0 (0)	2 (8)	2 (4)
Diet only	0 (0)	1 (4)	1 (2)
Rosiglitazone	0 (0)	1 (4)	1 (2)
Sulphonylurea	2 (9)	9 (38)	11 (24)
Any other OAD	3 (14)	4 (17)	7 (15)
No metformin (combination of diet and other OADs)	17 (77)	7 (29)	24 (52)
^b^New users of OADs			
Yes	3 (14)	7 (29)	10 (22)
No	17 (77)	12 (50)	29 (63)
Unsure	2 (9)	5 (21)	7 (15)

CPRD, Clinical Practice Research Datalink; GPRD, General Practice Research Database; HCC, Heaptocellular Carcinoma; ICC Intrahepatic Cholangiocarcinoma ; OAD, oral diabetic agent.

a Studies may have multiple outcomes; therefore column percentages will not sum to 1.

b Based on whether clear description is given in methods.

Although none of the investigators were blinded to the aims of the review, detailed criteria for assessment of bias were produced in order to consider risks of bias for each study. The eight domains assessed for bias were: (i) outcome definition; (ii) exposure definition (including choice of comparator): (iii) control selection (case control studies only); (iv) consideration of HbA1c, BMI and other antidiabetic drugs as time-dependent confounders affected by previous treatment (Box 1); (v) adjustment for baseline (study entry) confounders (smoking, diabetes severity, age, gender); (vi) immortal time (cohort studies only); (vii) missing data; and (viii) censoring methods (cohort studies only). For each bias domain, pre-defined criteria allowed categorization into high, medium, low or unlikely risk of bias. Bias in study estimate occurs when aspects of the design or data analysis either induce or fail to eliminate non-causal imbalances in risk of cancer between those who are exposed or unexposed. How this may occur is dependent on the bias domain in question, and detailed criteria for each domain are presented in Supplementary Table 2 (available as Supplementary data at *IJE* online). Broadly, studies were considered at unlikely risk of bias in a particular domain if the design and analysis methods were unlikely to induce a systematic difference between risk of cancer between metformin users and non-users. Low risk meant that there was small possibility of bias but the potential magnitude of the bias was unlikely to materially affect the overall study conclusions. Medium and high risk of bias meant that there was potential for some or substantial effect of bias on the study estimate, respectively. Although the specific criteria for each bias domain may have left some room for subjectivity, they were developed and agreed in advance by R.F., K.B. and D.F. to make them as objective as possible.

**Table 2.** Adjustment method for key time-dependent confounders affected by previous treatment: case-control studies

Study name	HbA1c			BMI			Other diabetic medication		
	Adjusted for value before first exposure	Adjusted for value between exposure and index date^a^	Adjusted for value at index date^a^	Adjusted for value before first exposure	Adjusted for value between exposure and index date^a^	Adjusted for value at index date^a^	Adjusted for value before first exposure	Adjusted for value between exposure and index date^a^	Adjusted for value at index date^a^
Azoulay *et al.* (2011)^24^		✓			✓			✓	
Becker *et al.* (2013)^25^					✓			✓	
Bodmer *et al.* (2011)^29^		✓			✓			✓	
Bodmer *et al.* (2010)^30^		✓			✓			✓	
Bodmer *et al.* (2012) (Lung)^26^					✓			✓	
Bodmer *et al.* (2012) (Pancreatic)^28^					✓			✓	
Bodmer *et al.* (2012) (Colorectal)^27^					✓			✓	
Bosco *et al.* (2011)^31^									
Chaiteerakij *et a*l. (2013)^32^									
Dabrowski *et al.* (2013)^34^								✓	
Donadon *et al.* (2010)^35^			✓			✓			
Li *et al.* (2009)^37^					✓			✓	
Evans *et al.* (2005)^7^					✓				
Hassan *et al.* (2012)^38^									
Margel *et al.* (2013)^39^								✓	
Mazzone *et al.* (2012)^40^		✓			✓				
Monami *et al.* (2009)^42^			✓			✓		✓	
Monami *et al.* (2011)^41^				✓				✓	
Smiechowski *et al.* (2013)^43^	✓^b^	✓		✓^b^	✓		✓^b^	✓	
Wang *et al.* (2013)^44^									
Chen *et al.* (2013)^33^								✓	
Donadon *et al.* (2010)^36^						✓			

a Index date, time of cancer diagnosis/matched date for control.

b Sensitivity analysis assessed whether there was a difference between adjusting for covariates measured before exposure or any time between 1 year before exposure and index date.

Time-dependent confounders affected by previous treatment were considered as a separate domain in addition to baseline confounding, to highlight the difference between baseline confounding that could be easily adjusted for in a standard analysis, and the more subtle bias that may arise if time-dependent confounders affected by previous treatment are not correctly adjusted for. If studies omitted a particular confounder because they found it did not alter the estimate of metformin on cancer risk in a multivariable model, then they were not deemed to be at risk of bias due to its exclusion. However, the timing and accuracy of the confounder were still considered as sources of bias, since these aspects could have resulted in its incorrect omission.

Bias from outcome and exposure definition encompassed both misclassification bias, biases induced by timing of measurement, and whether the definitions may introduce selection bias. Potential bias induced by using time-varying exposure without consideration for the time needed for exposure to plausibly cause cancer, could be considered as inappropriate censoring or as inappropriate exposure definition; to avoid double counting, this was considered a censoring bias.

Some studies provided multiple estimates based on dose-response categories (13 studies), or differing comparators (five studies). In this situation, the main estimate used for our analyses was that deemed to be most comparable to other studies. For multiple estimates from a dose-response model, if an overall exposed vs non-exposed estimate was not presented (five studies), a middle category best representing a moderate level of exposure was taken.

### Meta-regression

As an exploratory analysis designed to investigate whether between-study heterogeneity in the observed effect of metformin could be explained by bias and other study level factors, a random effects meta-regression was performed. Separate regressions were performed for the five most common outcomes: all cancer, colorectal/bowel cancer, lung cancer, breast cancer and pancreatic cancer. Studies that reported only stratum-specific results (three studies) were each entered into a meta-analysis to generate a pooled estimate for that study, which was subsequently used in the meta-regression.

Study characteristics evaluated in the meta-regression were a subset of all available, based on a priori assumptions about which might have the largest impact on study estimates. Characteristics included were comparator exposure [diet only, other oral antidiabetic drugs (OADs), less metformin and no metformin (diet and other OADs combined)], bias in exposure definition, bias in outcome definition, bias from baseline adjustments, bias from time-dependent confounders, immortal time bias and whether the cohort were incident users of diabetic drugs. Zero was assigned to studies rated as unlikely or low in the bias assessment, and one to those rated medium or high. A binary variable was used to reduce sparsity. Backwards stepwise selection was used to identify which characteristics best explained study heterogeneity. A *P*-value cut-off of 0.4 was used due to small sample size and the large number of parameters in the full model. Analysis was conducted using STATA v14.

## Results

### Search and screening

**Figure 1.**
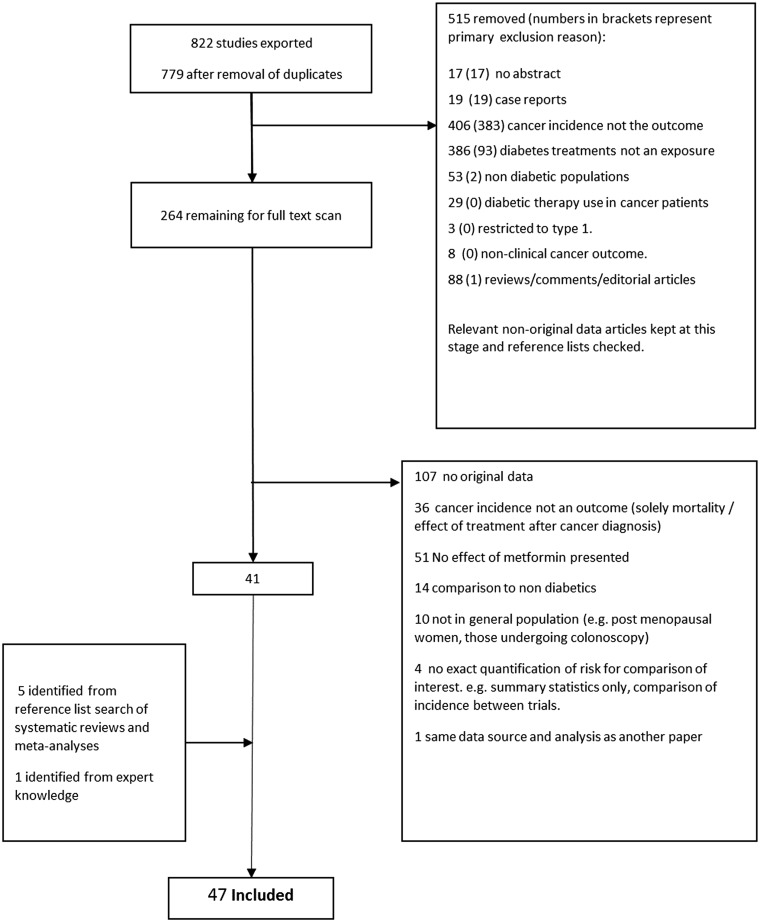
Flow chart of screening process detailing number of studies excluded at each stage and reason for exclusions.

The numbers of studies included/excluded at each stage of the process are presented in Figure 1. From an initial 822 references (779 after removal of duplicates), 46 studies were included in the final review. Full texts were available for all studies. The random sample of 76 studies independently screened by two researchers against the inclusion criteria resulted in a Cohen’s kappa of 0.79, and only a single initial disagreement over inclusion of a study; it was agreed on discussion that this study did meet the inclusion criteria. One article examined adverse event reports from two randomized controlled trials and so was technically not observational; however, it was included as it could be considered a retrospective cohort study with a trial-based data source. It did not adjust for age, but this exclusion criterion was waived since treatment was randomized.

Table 1 summarizes the data sources, outcomes, exposure definitions, timing of exposure measurements and comparator exposures used. More detailed study-level information is presented in Supplementary Table 3 (available as Supplementary data at *IJE* online).

**Table 3.** Adjustment method for key time-dependent confounders affected by previous treatment: cohort studies

Study name	HbA1c			BMI			Other diabetic medication		
	Adjusted for value at cohort entry (at time of or prior to first exposure)	Adjusted as a time- updated variable	Measured as an average of values at any point after exposure	Adjusted for value at cohort entry (at time of or prior to first exposure)	Adjusted as a time- updated variable	Measured as an average of values at any point after exposure	Adjusted for value at cohort entry (at time of or prior to first exposure)	Adjusted as a time- updated variable	Measured as an average of values at any point after exposure
Currie *et al.* (2009)^3^									
Currie *et al.* (2013)^47^	✓			✓					
Geraldine *et al.* (2012)^49^	✓			✓^a^					
^d^Home *et al.* (2010)^51^									
Hsieh *et al.* (2012)^8^									
Lai *et al.* (2012) (HCC)^52^									
Lai *et al.* (2012) (LUNG)^53^									
Lee *et al.* (2011)^54^									✓
Libby *et al.* (2009)^5^			✓			✓	✓^b^		
^e^Qiu *et al.* (2013)^59^									
Redaniel *et al.* (2012)^60^			✓	✓					
^e^Ruiter *et al.* (2012)^9^									
^e^Tsilidis *et al.* (2014)^61^				✓					
Yang *et al.* (2011)^63^	✓			✓					✓
Buchs and Silverman (2011)^45^									✓
Oliviera *et al.* (2008)^58^									
Hense *et al.* (2011)^50^				✓			✓		
Chiu *et al.* (2013)^46^									
Ferrara *et al.* (2011)^48^	✓							✓	
Lehman et al. (2012)55			✓						
Morden *et al.* (2011)^56^	✓^c^			✓^c^					
Neumann *et al.* (2011)^57^								✓	
Van Staa *et al.* (2012)^62^				✓				✓	
^e^Morgan *et al.* (2012)^64^	✓			✓					

a Weight used instead of BMI.

b Measured within 3 months/1 year of cohort entry (either side of first exposure).

c Diabetes complications used as proxy measures for severity.

d Treatment randomised so no adjustment necessary.

e Adjustment for use of other OADs not necessary as study looked at incident users of diabetes medications and censored at change in medication.

### Study characteristics

Of the 46 studies, 22 were case-control design^7^^,^^22–24^ and 24 were cohort studies.^3^^,^^5^^,^^8^^,^^9^^,^^45–63^ Data from electronic health records were used by 37 (80%) of the studies: most commonly, the UK’s Clinical Practice Research Datalink (CPRD) (13 studies) and the Taiwan National Health Insurance Claims Database (eight studies). As previously mentioned, one paper^51^ used data from two randomized controlled trials. The remaining eight (all case-control) collected data from a specific cancer or diabetes clinic.

A total of 22 studies (46%) defined exposure to metformin as any exposure, without considering overall duration. Three further studies refined this definition by requiring a minimum time period or number of prescriptions before an individual was considered exposed. Nine studies (20%) looked at monotherapy with metformin and 10 studies (22%) used total exposure to enable dose-response analyses. The remaining two studies looked at metformin in combination with specific OADs, with a comparator group that allowed the estimation of the effect of just metformin. The most frequently used comparator group was no metformin, used in 24 studies (52%). Use of sulphonylurea [another popular first-line oral agent; 11 studies (24%)] was also a common comparator.

There were 116 estimates presented for the effect of metformin on risk of cancer when considering separate estimates for different cancer sites. A total of 21 studies examined the outcome of all cancers excluding non-melanoma skin cancer (NMSC). Colorectal and/or bowel (14 studies) were the most common sites studied, followed by pancreas (13 studies), breast (13 studies), lung (12 studies) and prostate (11 studies). Other sites had less than 10 estimates each.

### Effect of metformin on cancer risk

**Figure 2.**
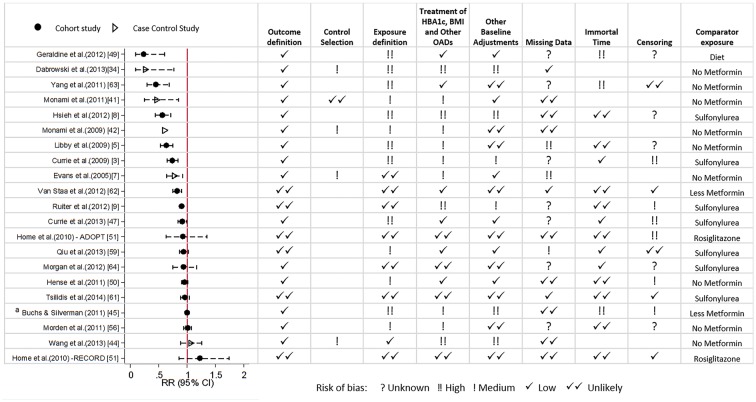
Estimated relative risk (odds ratio or hazard ratio) with 95% CI for the 21 studies examining use of metformin and risk of all cancers, and corresponding assessment of bias according to pre-specified criteria. ^a^ Represents the hazard ratio for cancer risk per one extra prescription of metformin.

**Figure 3.**
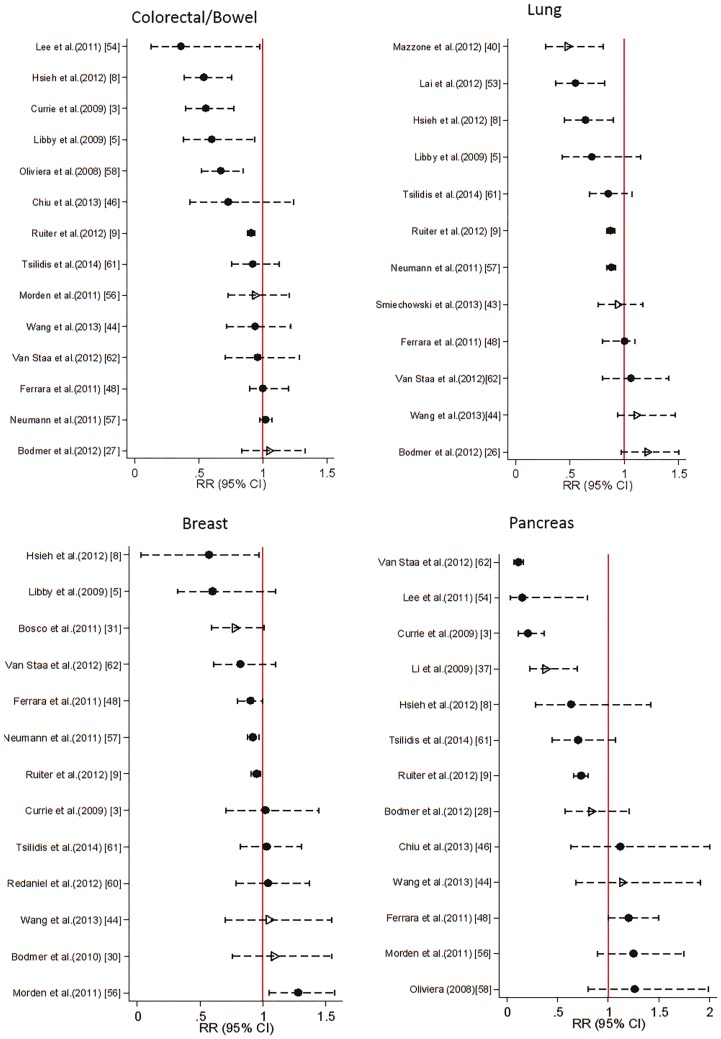
Estimated relative risk (odds ratio or hazard ratio) with 95% CI for 4 most commonly studied site specific cancers. Case control studies are represented by hollow triangle, Cohort studies by filled circles.

Figure 2 displays the study estimates and 95% confidence intervals (CIs) for relative risk [odds ratio (OR) or hazard ratio (HR)] of metformin use on incidence of all cancer. Estimates and 95% CIs for the four most commonly studied site-specific cancers are presented in Figure 3.

For all cancer, 18/21 studies estimated a protective effect of metformin, with 12/16 having upper confidence limits below 1. The magnitude of the effect estimates ranged from just a 0.04% reduction in risk^45^ to a 77% reduction in risk.^49^ For site-specific cancers, estimates were also highly variable across studies (Figure 3).

### Bias evaluation

Only three studies^51^^,^^61^^,^^62^ scored low or unlikely for risk of bias in all categories. One further study, which looked at lung cancer only, scored unlikely or low in all categories, except missing data^43^ where it was rated unknown. Three of these studies saw no evidence of an effect of metformin. One study estimated a modest protective effect of long-term use (> 60 months) in comparison with short-term use (0-6 months) with a hazard ration (HR) of 0.82 (0.75-0.90), but ultimately concluded that there was no evidence for a causal effect due to patterns of risk that were inconsistent with causality.^62^

Study-specific results of bias assessment for studies assessing all cancer as an outcome are displayed alongside risk estimates in Figure 2. Of the 12 studies that estimated a statistically significant protective effect of metformin, eleven had at least medium risk of bias in at least two domains; nine had medium or high risk of bias from exposure definition; and seven had medium or high risk of bias for treatment of HbA1c, BMI and other OADs. Bias assessments for all other studies are presented in Supplementary Table 4 (available as Supplementary data at *IJE* online).

**Table 4.** Parameter estimates from meta-regression models after backwards stepwise selection

		All cancer		Colorectal/Bowel			Lung		Breast			Pancreatic	
		Estimate 95% CI for effect on log risk ratio	*P*-value		Estimate 95% CI for effect on log risk ratio	*P*-value	Estimate 95% CI for effect on log risk ratio	*P*-value		Estimate 95% CI for effect on log risk ratio	*P*-value	*Estimate 95% CI for effect on log risk ratio	P value
Comparator group	No metformin	0 (ref)			0 (ref)		0 (ref)			0 (ref)		0 (ref)	
	Diet only	−1.16 (−2.41, 0.10)	^d^0.217										
	Less metformin	0.14 (−0.28, 0.55)			−0.04 (−0.43 , 0.34)	^d^0.386	0.05 (−0.34, 0.44)	^d^0.107		−0.37 (−0.81, 0.07)	^d^0.625	−1.66 (−3.30, −0.01)	0.004^d^
	Other OAD	0.10 (−0.19, 0.38)			−0.09 (−0.24 , 0.05)		−0.15 (−0.31, 0.02)			−0.22 (−0.41, -0.02)		0.36 (−1.10, 1.83)	
Bias from exposure definition	Low risk	0 (ref)			0 (ref)		0 (ref)					0 (ref)	
	High risk	−0.16 (−0.43, 0.10)	0.208		−0.40 (−0.58, −0.21)	0.001	−0.44 (−0.72, -0.17)	0.007				−0.84 (−1.74, −0.06)	0.06
Bias from outcome definition	Low risk						0 (ref)					0 (ref)	
	High risk						−0.17 (−0.48, 0.14)	0.234				−0.58 (−1.75, 0.59)	0.238
Immortal time bias	Low risk											0 (ref)	
	High risk											0.96 (0.03, 1.90)	
Bias from time- dependent confounding	Low risk				0 (ref)					0 (ref)		0 (ref)	0.046
	High risk				0.11 (−0.06, −0.28)	0.171				0.22 (0, 0.44)	0.047	0.92 (−0.13, 1.96)	0.071
Bias from baseline confounding	Low risk				0 (ref)					0 (ref)		0 (ref)	
	High risk				−0.12 (−0.18, −0.06)	0.002				−0.22 (−0.46, 0.02)	0.069	−1.05 (−2.01, −0.10)	0.037
Incident users	Yes						0 (ref)			0 (ref)		0 (ref)	
	No						0.18 (−0.14, 0.5)	0.218		−0.25 (−0.5, −0.01)	0.041	1.28 (−0.20, 2.76)	0.074
Constant		−0.15 (−0.43, −0.13)	0.269		0.00 (−0.14, 0.15)	0.954	0.01 (−0.16, 0.18)	0.892		0.17 (−0.08, 0.42)	0.155	−0.55 (−2.07, 0.97)	0.371
^a^I squared		85.21%			0%		0%			3.5%		9.5%	
^b^Adjusted R^2^		−20.32%			100.00%		100%			−190%		99.97%	
^c^Tau^2^		0.046			0		0			0.000305		0.000156	

Estimate represents the expected change in the log risk ratio (either HR or OR, depending on analysis method) for the effect of metformin on cancer, for each study level predictor. For example, a study of metformin and lung cancer, in which there is high risk of bias from exposure definition, is estimated to have a log risk ratio 0.44 lower than a study not at risk of bias from exposure definition.

a I squared is the estimate of residual variation due to study heterogeneity,

b Adjusted R^2^ is the estimated proportion of between study variance explained by the covariates in the meta regression. This can be negative when the between study variation in the model is increased because of loss of degrees of freedom more than it is improved by the addition of the covariates.

c Tau^2^ is the estimate of the remaining between-study variance.

d Joint p value for test of all levels comparator group.

#### Time-dependent confounders affected by previous treatment (HbA1c, BMI other antidiabetics)

Only four studies were considered as unlikely to be affected by bias due to how HbA1c, BMI and other antidiabetic treatments were accounted for in the analysis. These studies considered exposure to metformin as fixed from baseline [‘intention to treat’ (ITT) principle], and had confounders measured immediately before baseline.

Only 16/47 studies included HbA1c as a confounder in the final model. Six further studies reported considering it as a potential confounder, but did not include it in their final model due to lack of statistical significance^3^^,^^45^ or because it did not alter the results of the multivariable model.^25^^,^^27^^,^^59^^,^^61^ All but one of these studies^61^ were still considered at risk since it was questionable whether the HbA1c used was representative of HbA1c at the time of starting treatment; 26 studies accounted for BMI in their final model. In most case-control studies, the measurement of HbA1c and BMI preceded the date of cancer diagnosis (or matched date for the control) but it was rarely clear where this occurred in relation to the measurement of exposure, and therefore the potential for these studies to have adjusted for factors on the causal pathway between metformin and cancer was high. For the cohort studies, most used BMI and HBA1c measurements at or close to the time of cohort entry, which therefore either preceded or coincided with exposure classification. None of the studies reviewed used time-updated values of either HbA1c or BMI, though some used averages across follow-up.

The appropriate adjustment for other antidiabetic drugs is dependent upon the exposure and comparator group definitions. In six of the cohort studies examined, adjustment for use of other diabetic drugs was not necessary.^8^^,^^9^^,^^51^^,^^59^^,^^61^^,^^64^ In the remaining studies, 22 accounted for OADs. Tables 2 (case-control) and 3 (cohort) detail which adjustments were made, and the timing of the measurement within the follow-up period for each study separately.

#### Other sources of bias

Exposure definition (*n* = 28) and baseline adjustments (*n* = 22) were the other most common reasons for medium or high risk of bias. The exposure definition was most likely to have introduced bias in case-control studies by having different time windows to measure exposure, meaning the overall chance of seeing individuals exposed to metformin is systematically different between the cases and controls. Bias was most often introduced into cohort studies because future information was used to inform exposure definition; 7/24 cohort studies were considered to have high risk of immortal time bias. In all, 22 studies were considered at risk of bias from confounding due to incomplete or inappropriate baseline adjustment because either the comparator used may have resulted in comparing patients at differing disease stages without adjustment for baseline disease severity, or measures of severity used in the adjustment could be on the causal pathway between exposure and outcome, therefore not correctly accounting for differences in disease severity that may have influenced choice of treatment at baseline.

In addition, 36 studies were considered at risk of bias due to not considering a latency period for cancer (outcome definition). Since the effect of this bias is probably small in magnitude, this was considered to be low risk. This was supported by the five studies that considered different latency periods in sensitivity analyses, concluding that estimates did not differ substantially.^9^^,^^24^^,^^43^^,^^60^^,^^61^

Many studies were considered as at unknown risk of bias for censoring (12/24 cohort studies) and missing data (16 studies) due to a lack of information. Particularly for censoring, few cohort studies reported the numbers lost to follow-up or for what reason. Four studies were rated medium or high for risk of bias from missing data, three of these because the missing indicator method was used, which will increase the risk of residual confounding.^65^ With these three studies having > 20% missing data, the effect of residual confounding could be large.

### Meta-regression

Table 4 presents estimates and model diagnostics for the final meta-regression models obtained. For the outcome of all cancer, after backwards stepwise selection, the study level predictors that remained in the model were comparator group and exposure definition. The model estimated that using a comparator group of diet, as opposed to no metformin, made metformin appear more protective, whereas using other OADs or less metformin as a reference group made metformin appear less protective. However, this model was estimated to still have 85% residual variation due to heterogeneity. The comparator group was also retained in the models for site-specific cancers; however in the models for colorectal, lung and breast cancers, using other OADs as the comparator was estimated to make metformin appear more protective.

The strongest predictor of heterogeneity for studies of lung cancer was risk of bias from exposure definition which, if present, was estimated to reduce the log risk ratio by 0.44, 95% CI (0.17, 0.72) *P* = 0.007, making metformin appear more protective. For breast cancer, the strongest predictor was use of an incident user cohort, which made metformin look less protective. This predictor was also identified for studies of lung and pancreatic cancer, but the estimates had much less precision. Presence of both time-dependent and baseline confounding was also estimated to influence study heterogeneity for breast cancer, with presence of these biases estimated to have equal and opposite effects on the log risk ratio. For colorectal cancer, the strongest predictor was biased exposure definition, which was estimated to make metformin appear more protective.

## Discussion

The 46 studies examined in this review did not provide consistent evidence to support a protective effect of metformin on risk of cancer. Two of three studies with low or unlikely risk of bias for all categories had estimates consistent with no effect of metformin. The third study had an estimate consistent with a moderate protective effect; however this study included many analyses, and also reported that when comparing metformin exposure with other classes of oral antidiabetics, the risk of cancer did not differ between drugs. The authors also found that the incidence rates of cancer were higher in the first 3 months after therapy initiation, which they suggested might be due to detection bias, which would also explain why longer exposure appears protective when compared with the first 6 months of therapy.

**Figure 4.**
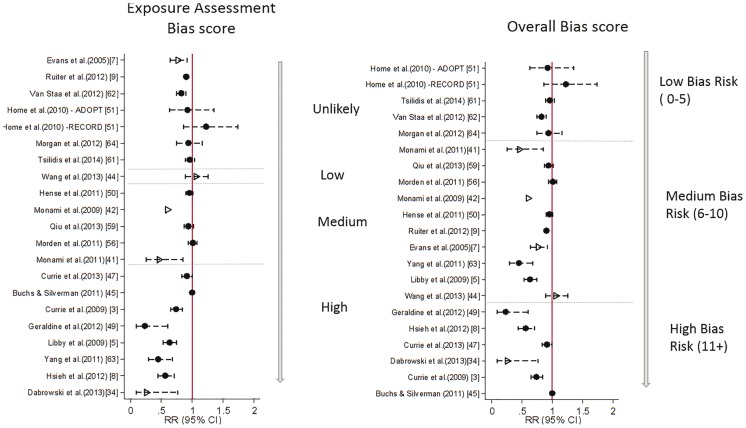
Estimates of relative risk of cancer from metformin use, ordered by risk of bias from exposure assessment only (left) and by overall risk of bias (right). Overall bias score is sum of bias risk over all domains, with unlikely = 0, low = 1, medium = 2, high = 3. Case control studies are represented by hollow triangle, Cohort studies by filled circle.

The estimates of effect reported across the 46 studies were highly variable for all outcomes studied. Many studies were at high risk of bias from exposure definition which, for reasons already outlined by Suissa and Azoulay,^10^ can have a large effect on estimates of risk. Within studies considered to be at low or unlikely risk of such bias, effect estimates were closer to the null but there was still variation in point estimates, albeit with some wide confidence intervals. Figure 4 displays the study estimates from Figure 2 ordered by risk of bias from exposure definition (left) to demonstrate this. It is possible that confounding by disease severity, and in particular confounding from time-dependent variables affected by previous treatment, could partly explain the remaining heterogeneity in observed estimates. By assigning values of 0, 1, 2 and 3 to unlikely, low, medium and high risk, respectively, and summing over all domains, an overall bias score was calculated. When ordered by this score [Figure 4 (right)] it is clear that heterogeneity increases as risk of bias increases, and the strongest protective effects are from those studies with the highest risk of bias overall.

The bias evaluation performed was detailed and thorough, and every effort was made to agree in advance the criteria for risk of bias in each of the eight domains examined. However, as in all studies of this kind, it was not possible to eliminate all subjectivity from this process.

**Figure 5.**
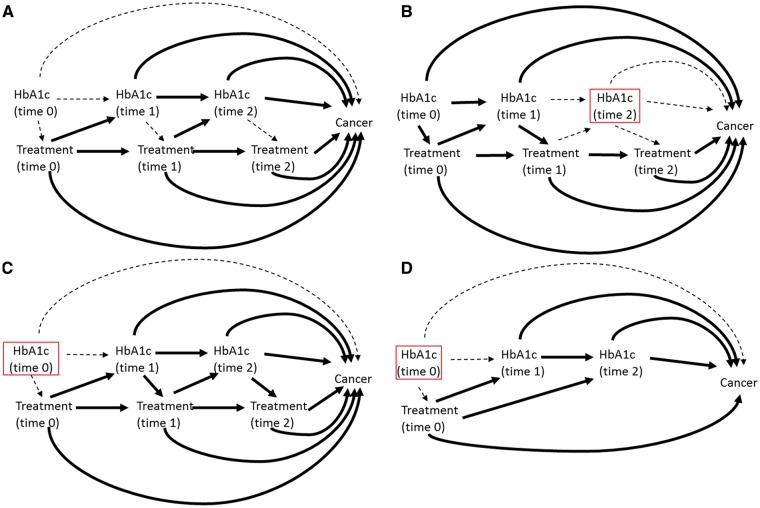
Directed Acyclic Graphs (DAGs) to represent estimated causal pathways for A) the desired total causal effect of treatment on cancer risk, and B)-D) the estimated effect under different methods of adjustment for time dependent confounders affected by prior treatment. Box indicates adjustment. Dotted line represents causal associations that are present but not included in the desired/estimated effect. A Solid lines represent the pathways needed to estimate the total causal effect of time varying treatment on cancer. B HbA1c measured at a single time point during the measurement window (usually the most recent value). Exposure may be time updated or assumed fixed from cohort entry. Solid line represents the pathways included in the estimate of effect under this approach. C HbA1c measured once at/before cohort entry, exposure modelled as time varying. Solid line represents the pathways included in the estimate of effect under this approach. D Exposure is assigned at cohort entry and assumed fixed (Intention to treat (ITT) principle), HbA1c measured once at/before cohort entry. Solid line represents the pathways included in the estimate of effect under this approach.

Figure 5A represents the total causal effect of metformin use on cancer risk that we wish to estimate in a simple example where we assume HbA1c is the only time-dependent confounder affected by previous treatment (as previously defined in Box 1). Figure 5 B, C and D illustrates the causal pathways that are actually being estimated under the three approaches most commonly used in the studies examined in this review. In Figure 5B, studies adjust for HbA1c but the measurement is taken any time during follow-up, which may result in ‘adjusting out’ any effect of metformin that is mediated through HbA1c. In Figure 5C, because treatment may change after baseline, the single adjustment at time 0 may lead to residual confounding by post-baseline HbA1c. In Figure 5D, the fixing of exposure from baseline removes the issue of time-dependent confounding and therefore allows the total effect of exposure on cancer to be estimated, but typically estimates an ITT effect only, which may not be appropriate given that patients are unlikely to adhere to a single treatment throughout follow-up. One study adjusted for non-adherence^61^ using a method that produces an unbiased estimate if there are no unmeasured confounders of the association between non-adherence and outcome,^66^ but the validity of this assumption is questionable. This approach is also limited by considering comparisons between active drugs only. When applied and analysed carefully, it will give an unbiased estimate of the effect of initiating metformin compared with initiating (as an example) sulphonylurea on development of cancer. However, this is not necessarily equivalent to estimating causal pharmacological effect of metformin use on cancer incidence and may be inappropriate if the comparator in question may itself affect risk of cancer. Most studies with low risk of other biases used the approach outlines in 5D. The lack of variation in how time-dependent confounders were adjusted for in these studies mean that it is not possible with the current literature alone to assess whether there is a meaningful impact of time-dependent confounders affected by previous treatment on the estimated effect of metformin on cancer risk.

In order to estimate the causal pharmacological effect of metformin on risk of cancer, the ideal would be to emulate a randomized controlled trial where patients are randomized to either metformin or diet only. This would involve comparison of patients initiating metformin with those controlling their disease by diet only, and correctly adjusting for disease severity at time of initiation while maintaining the effect of previous treatment on future disease severity measures. Causal methodology has been successfully used in other areas to overcome issues with time-dependent confounders affected by previous treatment,^67^^,^^68^ and could be applied to this question as a valuable addition to the current literature.

These causal inference methods (marginal structural models with inverse probability of treatment weighting or the g-computation formula) may be required to fully guard against some of the potential biases we identified, notably time-dependent confounders affected by previous treatment.^69^ However, even with standard analytical approaches, careful study design and analysis can minimize the risk of bias being introduced. For example, it is desirable to clearly identify incident users of oral diabetes medications, ideally in patients with newly diagnosed diabetes, and to ensure that important confounders such as HbA1c, BMI and other disease severity measures are recorded and adjusted for at study entry–either before or at the time of medication initiation. This will ensure that disease severity is broadly balanced at study entry, and that the effect of medication on future values of important covariates is not eliminated. In addition, if medication use is not assumed to be fixed from baseline, then it is important to classify time before first exposure as unexposed in order to avoid introducing immortal time bias. Secondary analyses to look at effects of cumulative exposure, and sensitivity analysis with exclusion of periods in which un-diagnosed cancer may be affecting probability of treatment, would also be advisable to establish whether observed associations are likely to be causal.

This review has systematically identified and assessed the existing literature on the pharmacoepidemiological question of metformin use and cancer risk. The search identified a large number of studies from varying countries and journals, and the inclusion criteria were shown to have good reliability between raters. Only one database was used in the search, and therefore some relevant literature may have been omitted from the review. However, by searching reference lists of other meta-analyses and systematic reviews, the majority of studies will still have been identified. Since performing the original search, it is likely that new studies will also have been published on the topic; however, a brief updated search did not identify any new studies that used methods substantially different from those covered in this review, though one study used slightly more sophisticated methods to deal with baseline confounding by indication.^70^ The meta-regression aimed to establish whether any of the potential sources of bias could explain the heterogeneity in risk estimates. A comparator group was selected for the final model in all analyses as a predictor of heterogeneity, but the direction of effect was inconsistent between models. Use of a non-incident user cohort was also identified in three models as a predictor of heterogeneity, but estimates of how this would affect study results were imprecise.

The overall reliability of the meta-regression results is questionable. For all cancer there were 21 studies contributing to this analysis, and even after selecting only key study level predictors, there were nine parameters in the initial model. The analysis was likely underpowered, and backward selection may not have produced reliable results. Additionally, many of these estimates lacked precision. For the site-specific cancers, since the sample sizes for the meta-regressions were smaller, these issues may be enhanced further and individual studies with extreme estimates are likely to have had a large influence. Furthermore for some biases, two high-risk studies could be rated as such for different reasons, which would bias the estimate in opposite directions, resulting in the bias appearing to have no effect overall. In addition, the ability to examine only published studies may itself introduce a publication bias which cannot be accounted for in a meta-regression. Also as previously mentioned, the bias evaluation could not be perfectly objective, which adds further uncertainty to any results of this analysis. Therefore, overall the results of this exploratory analysis should be interpreted cautiously.

Overall, the existing literature provides inconsistent answers to the question of metformin use and cancer risk in type 2 diabetes. Variation in design of studies and the potential for many kinds of bias make it difficult to explain the differences in risk estimates, particularly in terms of the potential impact of less easily detectable bias such as that from time-dependent confounders affected by previous treatment. It is likely that the largest protective effects that have been observed are a result of immortal time bias and other issues relating to how metformin use is defined. Studies without such biases tend to have estimates closer to the null, and whereas an effect of metformin use on risk of subsequent cancer in patients with type 2 diabetes cannot be excluded, the previously reported large protective associations are unlikely to be causal.

## Supplementary Data

Supplementary data are available at *IJE* online.

## Funding

This work was supported by the Medical Research Council London Hub for Trials Methodology Research [PhD studentship to R.F.], Wellcome Trust/Royal Society Sir Henry Dale Fellowship [107731/Z/15/Z to K.B.] and Wellcome Trust Senior Research Fellowship in Clinical Science [098504/Z/12/Z to L.S.].

**Conflict of interest**: There is no conflict of interest to report.

Key Messages

- Many existing observational studies investigating the effect of metformin use on cancer incidence in patients with type 2 diabetes have risk of bias.
- No studies to date have used appropriate statistical models to estimate the effect of time-varying treatment correctly controlling for time-dependent confounders which may be affected by previous treatment.
- Studies at lowest risk of bias do not support the hypothesis that metformin is protective against cancer.
- Previously reported large protective associations are unlikely to be causal.

## Supplementary Material

Supplementary Data
