# Metformin and cancer in type 2 diabetes: a systematic review and comprehensive bias evaluation

**DOI:** 10.1093/ije/dyx046

**Published:** 2017-04-12

**Authors:** Ruth E Farmer, Deborah Ford, Harriet J Forbes, Nishi Chaturvedi, Richard Kaplan, Liam Smeeth, Krishnan Bhaskaran

First published online: 12 December 2016, *Int J Epidemiol*, 2016, doi: https://doi.org/10.1093/ije/dyw275

The reported hazard ratios for two of the 46 papers included in this systematic review were incorrect. The estimates from Currie et al (2009) (reference 3) and Currie et al (2013) (reference 47) should have been inverted. This error affected some of the results reported in the abstract and the main text; the relevant data points on Figures 2, 3 and 4 and in the supplementary table; some of the data in Table 4; and one sentence in the discussion. All these items have now been corrected. The key results and conclusions were unaffected by this error.

