# Supplementary material for: Metformin and cancer in type 2 diabetes: a systematic review and comprehensive bias evaluation
Source: Int J Epidemiol. 2016 Dec 12;46(2):728–44. doi: 10.1093/ije/dyw275 (PMC5837266; doi:10.1093/ije/dyw275)
Supplement: Supplementary Data [file dyw275_supp.zip › ije-2015-12-1620-File012.docx]

**MOOSE Checklist**

From: Stroup DF, Berlin JA, Morton SC, et al (2000) Meta-analysis of observational studies in epidemiology: A proposal for reporting. JAMA 283:2008–2012. doi:10.1001/jama.283.15.2008.

|  | Reported on page | Comments |
| --- | --- | --- |
| **Reporting of background should include** | | |
| Problem definition | 3 |  |
| Hypothesis statement | 3 |  |
| Description of study outcome(s) | 3 | Final paragraph of intro |
| Type of exposure or intervention used | 3 | Final paragraph of intro |
| Type of study designs used | 3 | Final paragraph of intro |
| Study population | 3 | Final paragraph of intro |
| **Reporting of search strategy should include** | | |
| Qualifications of searchers (e.g. librarians and investigators) |  |  |
| Search strategy, including time period used in the synthesis and key words | 4 | Paragraph 1 |
| Effort to include all available studies, including contact with authors | 6 | Full text available for all studies needed for the full text screen. |
| Databases and registries searched | 4 | Paragraph 1 |
| Search software used, name and version, including special features used (e.g. explosion) | 4 & Supplementary | MEDLINE, through OVID. Full search terms in supplementary |
| Use of hand searching (e.g. reference lists of obtained articles) | 4 | Described in screening strategy |
| List of citations located and those excluded, including justification | Figure 1 | Full list of citations not given but flow chart of exclusions provided. |
| Method of addressing articles published in languages other than English | 4 | Non English language excluded |
| Method of handling abstracts and unpublished studies | 4 | Excluded |
| Description of any contact with authors | n/a | No contact with authors made |
| **Reporting of methods should include** | | |
| Description of relevance or appropriateness of studies assembled for assessing the hypothesis to be tested | 4 | Screening strategy – inclusion criteria described |
| Rationale for the selection and coding of data (e.g. sound clinical principles or convenience) | 3 (meta regression section) | For meta regression, selection of characteristics to include based on clinical and epidemiological principle, and coding of data based partly on convenience because of sample size, but this is based on statistical knowledge. |
| Documentation of how data were classified and coded (e.g. multiple raters, blinding and interrater reliability) | 4 & 5 & supplementary data | Data extraction described, and extraction table extract in supplementary |
| Assessment of confounding (e.g. comparability of cases and controls in studies where appropriate) | 4 & 5 & supplementary data | Bias domains assessed described. Full bias criteria available in supplementary. |
| Assessment of study quality, including blinding of quality assessors, stratification or regression on possible predictors of study results | 4 & 5 |  |
| Assessment of heterogeneity | 5 | Pre-planned meta regression due to expected study heterogeneity. |
| Description of statistical methods (e.g. complete description of fixed or random effects models, justification of whether the chosen models account for predictors of study results, dose-response models, or cumulative meta-analysis) in sufficient detail to be replicated | 5 | Random effects meta regression, study level predictors all defined and model fitting process described. |
| Provision of appropriate tables and graphics | Figure 1 | Figure 1 describes screening process. Otherwise N/A? |
| **Reporting of results should include** | | |
| Graphic summarizing individual study estimates and overall estimate | Figure 2 and 3 | Overall summary estimate not appropriate due to heterogeneity. |
| Table giving descriptive information for each study included | Table 1 & supplementary | Summary data in table 1, study level data in supplementary material |
| Results of sensitivity testing (e.g. subgroup analysis) | n/a | None performed, though results for site specific cancers also presented (figure 3), as are results by bias score (figure 4) |
| Indication of statistical uncertainty of findings | Table 4 | CIs and P-values presented in meta regression table |
| **Reporting of discussion should include** | | |
| Quantitative assessment of bias (e.g. publication bias) | Figure 4 | No funnel plots created for publication bias but studies examined by overall bias score. |
| Justification for exclusion (e.g. exclusion of non-English language citations) |  | No exclusions made that will have meant omitting relevant literature, with the exception of non English language. |
| Assessment of quality of included studies | 7-8 | Included in results as bias evaluation |
| **Reporting of conclusions should include** | | |
| Consideration of alternative explanations for observed results | 10&11 | General explanation of how time dependent confounding may have affected results, and discussion of meta regression results. |
| Generalization of the conclusions (i.e. appropriate for the data presented and within the domain of the literature review) | 10&11 |  |
| Guidelines for future research | 11 | Potential for causal inference methods to add to literature |
| Disclosure of funding source | 12 |  |
