# Supplementary material for: Metformin and cancer in type 2 diabetes: a systematic review and comprehensive bias evaluation
Source: Int J Epidemiol. 2016 Dec 12;46(2):728–44. doi: 10.1093/ije/dyw275 (PMC5837266; doi:10.1093/ije/dyw275)
Supplement: Supplementary Data [file dyw275_supp.zip › ije-2015-12-1620-File011.docx]

**Supplementary material:**

**Box 1: Full MEDLINE search terms**

1. Metformin/ or Insulin, Isophane/ or Insulin, Long-Acting/ or Insulin, Regular, Pork/ or Insulin/ or Insulin, Short-Acting/ or Insulin, Regular, Human/ or Hypoglycemic Agents/ or Thiazolidinediones/ or Sulfonylurea Compounds/ or (Metformin or (Insulin not ("insulin resistance" or "insulin receptor")) or Sulfonylurea* or Pioglit* or Thiazo*).ab.
2. Epidemiologic studies/ or case-control studies/ or cohort studies/ or exp clinical trial/ or meta-analysis/ or exp epidemiology/ or ("case control" or "case - control" or "cohort" or "follow up" or "longitudinal" or hazard or risk or rate or odds).mp.
3. Diabetes Mellitus, Type 2/dt
4. Diabetes Mellitus, Type 2/
5. neoplasms/ or exp neoplasms by histologic type/ or exp neoplasms by site/ or exp neoplasms, multiple primary/
6. (DIABETES and ("type" and ("2" or II))).ab.
7. 5 or (cancer or tumor or malig* or neoplas*).ab.
8. 3 and 7
9. (4 or 6) and 1 and 7
10. (8 or 9) and human/
11. 10 and 2
12. 11 not (Cell line/ or cell proliferation/ or genetic association/ or genotypes/ or animal/ or cytokine.mp. or chemokine.mp.)
13. limit 12 to english
14. obesity/ or weight loss/
15. 13 not 14

OR

1. (("anti diabet*" or "anti-diabet*" or Metformin or Sulfonylurea* or Pioglit* or Rosiglit* Thiazo* or antidiab* or (insulin not ("insulin resistance" or "insulin receptor" or "insulin-like" or "insulin like"))) and (cancer or (tumor not "tumor necrosis factor") or malig* or neoplas* or carcin*)).ti.
2. 16 and 2
3. 17 not 15
4. 18 not (Cell line/ or cell proliferation/ or genetic association/ or genotypes/ or animal/ or cytokine.mp. or chemokine.mp.)
5. limit 19 to English

Supplementary table 1a: Example data extraction – case control studies.

| Author (Year) | Azoulay (2011) | | Becker (2013) | | Bodmer (2011) | | Bodmer (2010) |
| --- | --- | --- | --- | --- | --- | --- | --- |
| Journal | Cancer Epidemiology Biomarkers & prevention | Gynecologic Oncology | | Gynecologic Oncology | | Diabetes care | |
| Data Source | GPRD | GPRD | | GPRD | | GPRD | |
| Cancer(s) of interest | Prostate | endometrial cancer | | Ovarian cancer | | Breast Cancer | |
| Within diabetic comparison the primary or a secondary analysis? | Primary | Secondary | | Secondary | | Primary | |
| Base cohort | Inclusions: Males aged >40, who are new users of OADs between 1988-2009 (1st ever prescription record and at least 12m of prior follow-up before this) Exclusions: those with insulin prior to first OAD or <1 year of OAD before index date | Inclusions: females below the age of 90 with > 3 years active history prior to cancer diagnosis/matched date  Exclusions: recorded code of hysterectomy more than 4 weeks prior to diagnosis, history of any other cancer (excl NMSC), alcoholism or HIV prior to index date. | | patients in GPRD (restricted to diabetics after case/control matching) | | Patients in GPRD with a diagnosis or diabetes and/or at least 1 prescription for an oral antidiabetic, aged between 30 and 79 years between 1994 and 2005. Exclusions: Patients on insulin only or with < 3 years of recorded history before diagnosis/first prescription. Patients with alcoholism and women with gestational diabetes anywhere in their follow up. | |
| Base cohort entry | Date of first OAD prescription | Date of entry into CPRD | | start of follow up | | Earliest of diagnosis or first OAD prescription. | |
| Base cohort exit | Earliest of: prostate cancer, death, end of follow-up end of 2009 | Cancer diagnosis/same date for matched control | | Cancer diagnosis/same date for matched control | | Cancer diagnosis/same date for matched control | |
| Cases | Prostate cancer, ascertained using algorithm incorporating relevant diagnostic, treatment and procedure codes | fist time diagnosis of endometrial cancer between 1995 and June 2012 | | First time diagnosis of ovarian cancer below 90 years of age between 1995 and 2009, with no history of any other cancer (except NMSC), alcoholism or HIV before diagnosis. Must have > 3 years history prior to index date | | women who had, after their first prescription for an oral antidiabetic drug, a recorded first time diagnosis of invasive breast cancer or carcinoma in situ that was followed by surgery, radiation, chemo, antiestrogen therapy or a combination of the above. (This would suggest that cancer before drug but after diagnosis is excluded? There are cases and controls in table 2 under "none" category for prescription so those who are never exposed to therapy must be included somehow. Potentially have excluded patients who got cancer before they started their drug, rather than including them as a case exposed to "no drug"? ) | |
| Controls | Alive, under follow-up and prostate-cancer free at time of index case diagnosis | matched from base population applying same exclusion criteria | | Patients with no diagnosis of ovarian cancer from base population. Same exclusion criteria as cases. In particular - no history of neither unilateral nor bilateral overactomy and no history of ovarian biopsy or cystectomy. | | free of any cancer diagnosis before the index date and have had a prescription for antidiabetic therapy before index date (unless they never have therapy?) | |
| Controls per case | 10 | up to 6 | | up to 6 controls selected at random | | up to 4 controls selected at random | |
| Matching factors | year or birth year, year of cohort entry, follow up time | Calendar time, age, sex, practise, number of active years in GPRD. | | Year of birth, sex, general practice, number of years active history and same index date applied. | | Age, sex, practice, index date. | |
| Exposure | 1: Any prescription of metformin in the period between cohort entry and 1 year before index date  2: Number of prescriptions for metformin in same period based on quartile distribution of that in controls | Use of metformin at least 2 years prior to index date. Short duration (1-24 prescriptions) long duration (25+) | | Exposure levels based on recorded number of prescriptions for oral antidiabetics before index date:  short tem 1-9 prescriptions  medium term 10-29  long term: >30 prescriptions  (defined to have equal numbers in groups) | | Exposure levels based on recorded number of prescriptions for oral antidiabetics before index date:  short tem 1-9 prescriptions  medium term 10-39  long term: >40 prescriptions  (defined to have equal numbers in groups) | |
| Exposure comparison | No metformin (so by definition, use of any other OAD) | No metformin but could be using other OADs | | No metformin but could be using other OADs | | No metformin but could be using other OADs | |
| Statistical analysis method used | Conditional logistic regression | Conditional logistic regression | | Conditional logistic regression , but not clear for within diabetic comparison as not matched on diabetes status? | | Conditional logistic regression | |
| Adjustments made & when measured | All measured prior to index date: Haemoglobin A1c (HbA1c; last measurement prior to index date), excessive alcohol use, obesity (BMI 30), smoking (ever vs. never), lower urinary tract symptoms (defined as either a diagnosis for benign prostatic hyperplasia or prostatitis and/or prescription for finasteride or dutasteride), previous cancer (other than non-melanoma skin cancer), and previous use of nonsteroidal anti-inflammatory drugs (NSAID), antihypertensive drugs, and statins. Further adjusted for ever use of other antidiabetic agents. | Use of other OADS (separated as metformin, sulfs, TZD and insulin, classified as never, short term, long term) BMI smoking and diabetes duration. | | bmi, smoking(non/current/past) prior use of estrogens and/or other oral contraceptive, history of hysterectomy > 3 years before index date, endometriosis and or polycystic ovaries, hba1c level (last before index date) , diabetes duration use of sulfs, use of insulin | | Use of prandial glucose regulators, acarbosem, estrogens, smoking, BMI, diabetes duration and a1c and also use of sulfs, thiazo and insulin. All measured as any time before index date (BMI time not specified) a1c was the last measurement before index date. | |
| Sensitivity analysis performed? | 1. Excluding first 2 years prior to index date instead of just 1.  2. Exclude controls diagnosed with prostate cancer within one year after being excluded in a risk set 3. Additional adjustment for PSA screening intensity.  4. Restricted to cases and matched controls with at least 5 years of medical follow up prior to index date. | restrict to cases with recorded hysterectomy, chemo, radiotherapy or specific oncology codes after index date, Analysis within diabetics only (which is the one reported in this table) | | none for the diabetic only analysis | | none described | |
| N (analysed) | 739 cases , 7359 controls | 291 cases 1746 controls (in diabetic analysis) | | 85 cases 480 controls | | 305 cases 1153 controls | |
| Average follow up time | Cases: 4.7 years Controls: 4.7 years | Minimum 3 years. Matched so there will be no imbalance | | not reported in diabetic only group | | not reported (minimum 3 years) | |
| Basic demographics? Age, gender split (if applicable)? | Mean age at index date: 74.1 (100% Male) | not reported in diabetic only group | | not reported in diabetic only group | | controls: (%) <40: 0.6% 40-49: 4.9% 50-59: 14.5% 60-69: 31.2% 70-79: 38.6% >=80: 10.2%  cases 1.6 , 4.6, 13.1, 32.5, 37, 11.2 | |
| Main reported results | Never vs ever exposure to metformin adjusted RR (risk ratio?) 1.23 (0.99 - 1.52).   Never vs different numbers of prescriptions: 1-7: 1.05 (0.80-1.37)  7-18: 1.29 (0.99 - 1.69)  18-36: 1.37 (1.04-1.81)  >36: 1.40 (1.03 - 1.89) | ORs for Metformin no of prescriptions vs no prior use: 1-24: 0.88 (0.61 - 1.25)  25+: 0.88 (0.58 - 1.32) | | Reference group: no prior use of metformin:  1-9 prescriptions: 0.59 (0.25-1.41) 10-29: 0.38 (0.14-0.97) >30: 0.38 (0.15-0.94)   NOTE - number of controls using metformin is larger in the restricted to diabetes table - not sure how this is possible? | | reference group: no prior use of metformin:  1-9 prescriptions: 1.20 (0.82-1.78) 10-39: 1.09 (0.76-1.55) >40: 0.44 (0.24-0.82) | |

Supplementary Table 1b: Example data extraction - Cohort studies. Full extraction for all studies available on request

| Author (Year) | Currie (2009) | Currie (2013) | Geraldine (2012) | Home (2010) |
| --- | --- | --- | --- | --- |
| Journal | Diabetologia | J Clin Endocrinol Metab | Diabetes Research and Clinical Practice | Diabetologia |
| Data Source | The Health Information Network (THIN) | CPRD (formerly GPRD) | Intego database - Belgian primary care database | ADOPT and RECORD clinical trials |
| Cancer(S) Of Interest | Any solid tumour, plus breast colon, pancreas and prostate individually | All cancer (not primary end point) | All cancer not including NMSC. Secondary outcomes of haematolymphopoetic, colorectal, lung & bronchus, urinary tract, breast and prostate. | All cancer not including NMSC |
| Within Diabetic Comparison The Primary Or A Secondary Analysis? | primary | primary | secondary | Primary |
| Cohort Definition | Diagnosis of diabetes presenting after 40 years of age, with at least 6 or more sequential prescriptions for an OHA, with first treatment after the year 2000, with no causes of secondary diabetes recorded. | Incident cases of diabetes from January 2000, as defined by either 1. More than one diagnosis code for T2DM. 2. One code not specific to diabetes plus a prescription or 3. At least 2 prescriptions. Must be at least 180 days from beginning of follow up to first record (to make sure incident diagnoses) | Patients with t2 diabetes within the database - identified as aged over 30 when diagnosed, for whom secondary causes of diabetes were not recorded and were not recorded as type 1. | Members of clinical trial (inclusion criteria in trial publications). |
| Cohort Entry | date of treatment initiation | first record of diabetes/prescription | date of registration | trial entry |
| Cohort Exit | switching to next treatment (and thus entering a different cohort exposure group) last record, cancer or death/transfer out | Date of outcome, date of switching regimes, or at final recorded data. | Cancer, death , end of registration or end of study period (2008) | cancer event, end of trial or loss to follow up |
| Exposure | 5 exposure groups:  1 & 2: Newly initiated OHA monotherapy (6 month wash in of no OHA) with metformin or sulf respectively  3. Newly identified switch from OHA monotherapy with sulf or met to combined therapy with both but nothing else  4. Previous treatment with OHA newly starting on insulin. | Metformin monotherapy, sulfonylurea monotherapy, insulin monotherapy, metformin plus sulf and insulin plus metformin. As identified by at least 180 days on the same regimen. Patients can contribute to multiple groups if they switch, but no follow up time can overlap between groups. | 1. Ever exposed to metformin during the study period (1994 - 2008)  2. Patients on other treatment (insulin/sulf) only  3. diet only | Randomisation to metformin or rosiglitazone in addition to sulfonylurea. |
| Reference Group | metformin monotherapy | Metformin monotherapy | Diet only | Rosiglitazone (TZD) |
| Outcome | primary outcome: first occurrence of any sold tumour after cohort entry  secondary outcome: first cancer observed following treatment initiation (cohort entry) out of breast, pancreatic, colorectal or prostate | Cancer, including solid tumours and haematological forms, defined by readcodes in patient record | all malignancy not including NMSC - validated with a random sample that was 78% accurate | Malignancy reported as an SAE. |
| Statistical Analysis Method Used | cox PH with time varying exposure | cox PH with time varying exposure | cox PH | cox PH |
| Adjustments Made & When Measured | Age, gender, smoking status and history of prior cancer (other covariates of HbA1c, diabetes duration and weight added but not significant so presume removed again) all measured at baseline (date of first prescription) | Age, gender, SBP, HbA1c , total cholesterol, serum creatinine, bmi, smoking status, other risk factor management, duration of diabetes, prior history of cancer, LVD, microvascular disease, number of contacts with GP in year prior to index date, charlson comorbidity index all measured at baseline (nearest value to) . | measured at cohort entry - age, gender, weight and HBA1c | none - but allocation randomised |
| Sensitivity Analysis Performed? | none described | Secondary analyses used a) insulin instead of metformin monotherapy as reference. B) Stratified by tertiles of baseline HBA1c and baseline morbidity. | none described | none described |
| N (Analysed) | 62809 patients overall   met monotherapy: 31421 sulf mono: 7439 met + sulf: 13882 insulin based: 10067  44% of insulin group also had person time in group 3. 23% of group 3 also had person time in 1 or 2 | 105 123 total | 2173 on met, 771 on other and 1068 diet only | ADOPT: metformin 1454, rosiglitazone 1456  RECORD: metformin 1122, rosiglitazone: 1103 |
| Mean Follow Up Time | 1. Metformin mono: 71261 2. Sulf mono: 17553 3. Metformin sulf combined: 34909 4. insulin based: 28342  (person years) | 298, 530 person years total. Mean years per person 2.8 for metformin mono therapy, sulf monotherapy and metformin + sulf. 3.3 years for insulin and 3.5 years for insulin + metformin | from start of registration period : 5.42 years in metformin group, 5.62 years in other antidiabetic group and 4.11 years in diet only group | ADOPT: Metformin 4906 person years, Rosiglitazone 4954 person years glibenclamide 4244 person years RECORD: Sulf + randomised met: 6126 py, sulf and randomised rosiglitazone 6110 person years |
| Basic Demographics - Age, Gender distribution if reported | Mean Age (SD) %female:  1. 58.6 (14.6) 48.9 2. 70.00 (13.9) 45.1 3. 64.4 (12.4) 42.1 4. 63.7 (12.9) 44.6 | Mean age (SD) % male :  Metformin: 61.9 years 56.5% male Sulf: 60.7 yrs. 55.9% male met+sulf: 61.5 yrs. 60% male insulin: 61.3 yrs. 52.8% male met+insulin: 57.6 yrs. 57.3% male | Metformin group: 53% male , average age 61.9 (calculated as weighted average as presented in paper by gender)  other treatment group: 49% Male, average age 67.0 years diet only group: 47% Male, average age 63.5 years | ADOPT: mean age 57 +- 10 years, 57.7% men RECORD: 51.6% male, average age 58.4 |
| Main Reported Results | relative to metformin mono therapy for:   outcome of all solid tumours:  Sulf monotherapy : 1.36 (1.19 - 1.54) met and sulf: 1.08 (0.96-1.21) insulin based: 1.42 (1.27-1.60)  breast cancer (women only) sulf mono: 0.98 (0.69-1.41) met + sulf: 0.90 (0.67-1.21) insulin based: 1.07 (0.79-1.44)  colorectal:  sulf: 1.80 (1.26-2.53) met + sulf: 1.43 (1.05-1.94) insulin based: 1.69 (1.23-2.33)  pancreatic:  sulf: 4.95 (2.74 - 8.96) met + sulf: 0.38 (0.13-1.12) insulin based: 4.63 (2.64-8.10)  prostate:  sulf: 1.07 (0.76-1.49) met + sulf: 1.18 (0.89-1.57) insulin based: 1.10 (0.79-1.52) | With metformin monotherapy as reference. HR for all cancer types for:  sulfonylurea 1.097 (1.004 , 1.199) p=0.0410 Metformin + sulfonylurea 0.956 (0.877 , 1.043) p=0.3120 insulin 1.437 ( 1.234 , 1.647)<0.001 insulin + metformin 1.394 (1.179 , 1.651)p=0.001 | Stratified by gender:  HRs for…  FEMALES  Metformin vs diet group: 0.20 (0.03 - 1.64) other treatment vs diet group: 0.17 (0.01 - 2.026) MALES Metformin vs diet group: 0.24 (0.08 - 0.72)  other treatment vs diet group: 0.22 (0.06 - 0.82) | ADOPT:  Met vs Rosiglitazone HR: 0.92 (0.63 - 1.35) Met vs Glibenclamide 0.78 (0.53 - 1.14)  RECORD:  ITT met vs rosiglitazone 1.22 (0.86 - 1.74)  Per protocol met vs rosiglitazone 1.12 (0.75 - 1.68) |

**Supplementary table 2a: Bias assessment criteria case control studies**

| Case Definition | **Unlikely:** Well defined/validated diagnosis e.g. Read codes, hospital records etc. Somehow incorporates / accounts for latency period before which cancer is likely present but not diagnosed (i.e. outcome is measured at a time that is in the appropriate risk period relative to exposure).  **Low risk:** definition of cancer diagnosis with some potential for non differential misclassification.  **Medium risk:** Possible that outcome may occur at a time that is not in the appropriate risk period relative to exposure (e.g. No lag period applied to allow for latency of cancer)  **High risk:** Unclear or un-validated method of diagnosis with potential for differential misclassification between exposed and unexposed. Likely that outcome can occur at a time that is not in the appropriate risk period relative to exposure |
| --- | --- |
| Control Selection | **Unlikely:** Controls comparable to the cases.  **Low risk:** Some uncertainty over whether controls are representative of the population that produced the cases.  **Medium risk:** Some potential for controls to be systematically at more or less risk than cases.  **High risk:** controls are likely to systematically be at more or less risk than controls/ clear suggestion that controls are not representative of the case population. |
| Exposure Assessment | **Unlikely:** Well defined information e.g. prescription records, with level of exposure clearly taken into account in some way e.g. total dosage, number of prescriptions, average daily dosage, length of exposure, with at least 3 categories (low, medium, high). Measured at time that is representative of the relevant risk period for developing cancer and time period for exposure assessment is balanced between cases and controls. Choice of referent group made in consideration of confounding by indication  **Low risk:** As above but: Level of exposure incorporated only crudely e.g. Dichotomised only as low/high OR with some potential for choice of referent group to cause bias. OR Small risk of misclassification due to incomplete records on past exposure e.g. Non incident users.  **Medium risk:** Slightly unclear definition of exposure OR assessed by patient recall OR potential imbalance in exposure ascertainment window between cases and controls OR strong potential for referent group to cause bias from confounding by indication.  **High risk**: How exposure ascertained unknown OR not clearly defined OR exposure measured at time that would not represent the relevant risk period for developing cancer (e.g. Within 1 year of index date or after matched index date in controls) OR clear and large imbalance in exposure ascertainment window. |
| Treatment of potential time dependent confounders (HBA1c, BMI, BP, Other medication)  **Note**: if studies considered them as adjustments but did not include in final models based on lack of change to effect in their data then it was treated as if they had been included | **Unlikely:** Treated as time dependent confounders with appropriate methods (e.g. MSM’s with IPTW or g computation) used to appropriately adjust. OR Time invariant exposure with confounder measured within a window prior to but close enough to exposure for its value to accurately represent level at time of exposure. Confounders measured as continuous or with sufficient detail (at least 3 categories).  **Low: Fixed exposure** Adjusted for values measured before exposure but possibly a long time before actual exposure OR as unlikely, but with less detail in confounder measures OR Time varying exposure adjusted for baseline values of time dependent confounders.  **Medium:** confounder measured at time point **after** exposure OR measured before exposure as presence of other things that may indicate level of disease severity or weight (i.e. Less accurate). OR as low but only some of the key TDCs adjusted for.  **High:** None of the key TDCs considered in any way. . |
| Adjustment of other key confounders  **Note**: If studies considered them as adjustments but did not include in final models based on lack of change to effect in their data then it was treated as if they had been included but timing still considered. | **Unlikely:** Adjusted for minimum of age, gender, some measure of disease severity (e.g. duration, previous treatment, HBA1c) BMI and smoking status (for all cancer and site specific known to be associated with smoking) with several categories for each (at least 3) or continuous if appropriate. Measured at correct time – i.e. baseline if it has potential to change through follow up. Measured from accurate information e.g. computer records.  **Low:** Adjusted for all confounders but with less detail (e.g. Binary) OR adjustment for most of the minimum set with adequate detail and appropriate timing, but some variables from the minimum set omitted.  **Medium:** Adjusted for most of the minimum but with less detail (e.g. Binary) or severity measure of any detail level but measured after exposure assessment/as average through follow up/at index date OR measured from inaccurate data e.g. Recall.  **High:** Adjusted for age and gender only |
| Missing data | **Unlikely:** None or very low percentage of missing data, or appropriate missing data technique used). Sensitivity analysis performed to assess potential impact of missingness.  **Low:** Small amount of missing data (<15%) with no or inappropriate method applied to deal with it. **Medium:** Substantial missing data (15-25%) with no or inappropriate method used.  **High:** Large amount of missing data (>25 %) with no discussion /attempt to assess impact, or inappropriate method used. |

**Supplementary table 2b: Bias assessment criteria cohort studies**

| Outcome Assessment | **Unlikely:** Well defined/validated diagnosis e.g. Read codes, hospital records etc. Somehow incorporates / accounts for latency period before which cancer is likely present but not diagnosed (i.e. outcome is measured at a time that is in the appropriate risk period relative to exposure).  **Low risk:** definition of cancer diagnosis with potential for non differential misclassification.  **Medium risk:** Possible that outcome may occur at a time that is not in the appropriate risk period relative to exposure (e.g. No lag period applied to allow for latency of cancer)  **High risk:** Unclear or un-validated method of diagnosis with potential for differential misclassification between exposed and unexposed. Likely that outcomes will be occurring at a time that is not in the appropriate risk period relative to exposure. |
| --- | --- |
| Exposure Assessment | **Unlikely:** Well defined information e.g. prescription records, with level of exposure taken into account in a time dependent manner with at least 3 categories (low, medium, high / short term, medium term, long term/ current, past, never etc.) Or, if ITT analysis, minimum exposure period before being classified as “exposed” to ensure actually exposed. Choice of referent group made in consideration of confounding by indication, If cohort not incident users then past exposures should be measured and adjusted for.  **Low risk:** As above but: less detailed measurement of cumulative exposure in a time varying analysis OR no minimum exposure period in ITT analysis OR some potential for choice of referent group to cause bias if appropriate adjustments not made elsewhere. OR some potential for misclassification of exposure due to lacking information on exposure prior to cohort entry.  **Medium risk:** exposure assessed by patient recall OR strong potential for misclassification of exposure status due to lack of information about exposure prior to cohort entry (e.g. Assessing exposure in a small time interval (<6 months) around cohort entry in a prevalent user cohort). OR Strong potential for referent group to cause bias if appropriate adjustments not made.  **High risk**: How exposure ascertained unknown/not clearly defined OR Future information used to inform exposure status at baseline. |
| Treatment of potential time dependent confounders (HBA1c, BMI, BP, Other medication) | **Unlikely:** Treated as time dependent confounders with appropriate methods (e.g. MSM’s with IPTW or g computation) used to appropriately adjust. OR Time invariant exposure with confounder measured within a window prior to but close enough to exposure for its value to accurately represent level at time of exposure. Confounders measured as continuous or with sufficient detail (at least 3 categories).  **Low: Fixed exposure** Adjusted for values measured before exposure but possibly a long time before actual exposure OR as unlikely, but with less detail in confounder measures OR Time varying exposure adjusted for baseline values of time dependent confounders.  **Medium:** confounder measured at time point **after** exposure OR measured before exposure as presence of other things that may indicate level of disease severity or weight (i.e. Less accurate). OR as low but only some of the key TDCs adjusted for.  **High:** None of the key TDCs considered in any way. |
| Baseline Adjustments | **Unlikely:** Adjusted for minimum of age, gender, some measure of disease severity (e.g. duration, previous treatment, HBA1c) BMI and smoking status (for all cancer and site specific known to be associated with smoking) with several categories for each (at least 3) or continuous if appropriate. Measured at correct time – i.e. baseline if it has potential to change through follow up. Measured from accurate information e.g. computer records.  **Low:** Adjusted for all confounders but with less detail (e.g. Binary) OR adjustment for most of the minimum set with adequate detail and appropriate timing, but some variables from the minimum set omitted.  **Medium:** Adjusted for most of the minimum but with less detail (e.g. Binary) or severity measure of any detail level but measured after exposure assessment/as average through follow up/at index date OR measured from inaccurate data e.g. Recall.  **High:** Adjusted for age and gender only |
| Missing data | **Unlikely:** None or very low percentage of missing data, or appropriate missing data technique used for example Multiple imputation). Sensitivity analysis performed to assess potential impact of missingness.  **Low:** Small amount of missing data (<15%) with no or inappropriate method applied to deal with it. **Medium:** Substantial missing data (15-25%) with no or inappropriate method used.  **High:** Large amount of missing data (>25%) with no discussion /attempt to assess impact, or inappropriate method used. |
| Immortal Time | **Unlikely:** No follow up time included in which the event cannot occur by definition.  **Low:** Immortal time included but not differential between exposure status  **Medium:** differential immortal time between exposure groups but only likely to have small impact on results  **High:** Immortal time in one exposure group but not another which is likely to have a significant impact on results. |
| Censoring | **Unlikely:** No censoring/loss to follow up present OR Some kind of sensitivity analysis or appropriate method of adjustment (e.g. IPW) used to assess impact of censoring. Censoring other than at loss to follow up (e.g. at change in treatment) is done with appropriate consideration of timing for exposure and outcome (e.g. not censoring at exact date of treatment change and hence excluding cancer event in the following week) if not accounted for in definition of exposure/outcome.  **Low:** No specific method used to account for censoring but unlikely that censoring in study will have an impact on the results.  **Medium:** No adjustments/additional analysis where it is possible that the censoring may cause bias OR potential for residual confounding between reason for censoring and outcome even after censoring adjustment applied.  **High:** censoring present that will likely impact conclusions, with no discussion/analysis to assess impact. |

Supplementary table 3a: Basic study level information: case control studies

| **Case control studies** | | | | | | | | | | |
| --- | --- | --- | --- | --- | --- | --- | --- | --- | --- | --- |
| **Author** | **Cancer site** | **Primary Relative Risk Estimate (95% CI)** | **Data Source** | **Exposure Definition** | **Comparator** | **Simplified Exposure Definition** | **Exposure Measurement time** | **Simplified Reference group** | **Incident users** | **Follow up time** |
| **Azoulay (2011) [24]** | Prostate | 1.23 (0.99 , 1.52) | CPRD | Any prescription of metformin in the period between cohort entry and 1 year before index date | other OAD | Any Exposure | Single summary measure of exposure over entire follow up. | Any other OAD | Yes | Mean 4.7 years |
| **Becker (2013) [25]** | Ovarian/Endometrial | 0.88 (0.58 , 1.32) | CPRD | long duration (>25 prescriptions) of metformin at least 2 years prior to index date | no metformin | Total Exposure (Number of prescriptions/time on metformin) | Single summary measure of exposure over entire follow up. | No metformin | No | Minimum 3 years |
| **Bodmer (2010) [30]** | Breast | 1.09 (0.76 , 1.55) | CPRD | 10 - 39 prescriptions of metformin prior to index date | no metformin | Total Exposure (Number of prescriptions/time on metformin) | Single summary measure of exposure over entire follow up. | No metformin | No | Minimum 3 years |
| **Bodmer (2011) [29]** | Ovarian/Endometrial | 0.38 (0.14, 0.97) | CPRD | 10-29 prescriptions of metformin prior to index date | no metformin | Total Exposure (Number of prescriptions/time on metformin) | Single summary measure of exposure over entire follow up. | No metformin | No | Not reported |
| **Bodmer (2012) (Colorectal) [27]** | Colorectal/Bowel | 1.05 (0.84 , 1.33) | CPRD | 10-29 prescriptions of metformin prior to index date | no metformin | Total Exposure (Number of prescriptions/time on metformin) | Single summary measure of exposure over entire follow up. | No metformin | No | Minimum 3 years |
| **Bodmer (2012) (Lung) [26]** | Lung | 1.21 (0.97 , 1.50) | CPRD | 15 - 39 prescriptions of metformin prior to index date | no metformin | Total Exposure (Number of prescriptions/time on metformin) | Single summary measure of exposure over entire follow up. | No metformin | No | Minimum 3 years |
| **Bodmer (2012) (Pancreatic) [28]** | Pancreatic Cancer | 0.83 (0.57 , 1.21) | CPRD | > 30 prescriptions of metformin prior to index date (only result reported in diabetic only analysis) | no metformin | Total Exposure (Number of prescriptions/time on metformin) | Single summary measure of exposure over entire follow up. | No metformin | No | Not reported |
| **Bosco (2011) [31]** | Breast | 0.78 (0.59 , 1.01) | Danish Medical Registry | Minimum 1 year use of metformin | other OAD | Any exposure but minimum time/number of prescriptions needed) | Single summary measure of exposure over entire follow up. | Any other OAD | No | Not reported |
| **Chen (2013) [33]** | HCC/ICC | 0.79 (0.75 , 0.83) | Taiwan National Health Insurance Claims database | Ever use of metformin in 5 years prior to index date | no metformin | Any Exposure | Single summary measure of exposure over entire follow up. | No metformin | No | 5 years |
| **Chaiteerakij (2013) [32]** | HCC/ICC | 0.20 (0.10, 0.40) | Mayo Clinic, Rochester MN | Ever use of metformin in the previous year | no metformin | Any Exposure | Current use | No metformin | No | 1 year |
| **Dabrowski (2013) [34]** | All Cancer | 0.26 (0.09 , 0.77) | Diabetic outpatient clinic, Poland | use of metformin prior to index date | no metformin | Any Exposure | Single summary measure of exposure over entire follow up. | No metformin | No | Not reported |
| **Donadon (2010) [35]** | HCC/ICC | 0.15 (0.04 , 0.51) | Pordenone General Hospital, Italy | use of metformin at index date | sulfonylurea | Any Exposure | Current use | Sulfonylurea | No | Not reported |
| **Donadon (2010) - 2 [36]** | HCC/ICC | 0.15 (0.04 , 0.50) | Pordenone General Hospital, Italy | use of metformin at index date | sulfonylurea | Any Exposure | Current use | Sulfonylurea | No | Not reported |
| **Li (2009) [37]** | Pancreatic Cancer | 0.38 (0.22 , 0.69) | University of Texas MD Anderson Cancer Centre. (MDACC) | Self-reported ever use of metformin prior to index date | no metformin | Any Exposure | Single summary measure of exposure over entire follow up. | No metformin | No | Not reported |
| **Evans (2005) [7]** | All Cancer | 0.77 (0.64 , 0.92) | Diabetes Audit Research Tayside (DARTS) and linked dispensed prescription database (MEMO) | Any metformin between 1993 and index date | no metformin | Any Exposure | Single summary measure of exposure over entire follow up. | No metformin | Not sure | Not reported |
| **Hassan (2012) [38]** | HCC/ICC | 0.30 (0.20 , 0.60) | University of Texas MD Anderson Cancer Centre. (MDACC) | Self-reported ever use of metformin prior to index date | no metformin | Any Exposure | Single summary measure of exposure over entire follow up. | No metformin | No | Not reported |
| **Margel (2013) [39]** | Prostate | 0.95 (0.85-1.07) | Ontario Diabetes Database, Ontario Cancer Registry and Ontario Drug Benefit Database | Ever use of metformin prior to index date | no metformin | Any Exposure | Single summary measure of exposure over entire follow up. | No metformin | Yes | Mean 2.9 years |
| **Mazzone (2012) [40]** | Lung | 0.48 (0.28 , 0.81) | Diabetic Clinic, Cleveland USA | Metformin monotherapy only before index date | neither metformin nor thiazodaline used prior to index date | Monotherapy with metformin | Single summary measure of exposure over entire follow up. | No metformin | No | not reported |
| **Monami (2009) [42]** | All Cancer | 0.60 (NO CI reported) | Diabetic outpatient clinic, University of Florence. | Any exposure to metformin in 10 years prior to index date | no metformin | Any Exposure | Single summary measure of exposure over entire follow up. | No metformin | No | Mean 6.5 years |
| **Monami (2011) [41]** | All Cancer | 0.46 (0.25 , 0.85) | Diabetic outpatient clinic, University of Florence. | Ever exposure to metformin after start of insulin and before index date | no metformin | Any Exposure | Single summary measure of exposure over entire follow up. | No metformin | No | Mean 6.3 years |
| **Smiechowski (2013) [43]** | Lung | 0.94 (0.76 , 1.17) | CPRD | Any prescription of metformin in the period between cohort entry (first ever prescription) and 1 year before index date | other OAD | Any Exposure | Single summary measure of exposure over entire follow up. | No metformin | Yes | Mean 5 years |
| **Wang (2013) [44]** | All Cancer | 1.06 (0.89 , 1.26) | Taiwan National Health Insurance Claims database | Ever use of metformin prior to index date | no metformin | Any Exposure | Single summary measure of exposure over entire follow up. | No metformin | Not sure | Not reported |
|  | Breast | 1.05 (0.70 , 1.55) |  |  |  |  |  |  |  |  |
|  | Colorectal/Bowel | 0.94 (0.73 , 1.21) |  |  |  |  |  |  |  |  |
|  | Lung | 1.11 (0.94 , 1.47) |  |  |  |  |  |  |  |  |
|  | Pancreas | 1.14 (0.68 , 1.91) |  |  |  |  |  |  |  |  |
|  | Prostate | 0.94 (0.61 , 1.46) |  |  |  |  |  |  |  |  |
|  | Stomach | 1.62 (0.99 , 2.64) |  |  |  |  |  |  |  |  |

Supplementary table 3b: Basic study level information: cohort studies

| **Cohort Studies** | | | | | | | | | | |
| --- | --- | --- | --- | --- | --- | --- | --- | --- | --- | --- |
| **Author** | **Cancer site** | **Primary Relative Risk Estimate (95% CI)** | **Data Source** | **Exposure Definition** | **Comparator** | **Simplified Exposure Definition** | **Exposure Measurement time** | **Simplified Reference group** | **Incident users** | **Follow up time** |
| **Buchs (2011) [45]** | All Cancer | 1.00 (0.99 , 1.00) | Maccabi Healthcare Services (MHS) computerised databases | Cumulative use of metformin between 2003 & 2007 | 1 less prescription for metformin | Total Exposure (Number of prescriptions/time on metformin) | Single summary measure of exposure over entire follow up. | Less exposure | No | Mean 4.5 years |
| **Chiu (2013) [46]** | Colorectal/  Bowel | 0.73 (0.43 , 1.24) | Taiwan National Health Insurance Claims database | Any exposure to metformin during the study period | no metformin | Any Exposure | Single summary measure of exposure over entire follow up. | No metformin | Not sure | Not reported |
|  | Oesophagus | 1.33 (0.49 , 3.59) |  |  |  |  |  |  |  |  |
|  | Liver | 0.98 (0.74 , 1.29) |  |  |  |  |  |  |  |  |
|  | Pancreas | 1.12 (0.63 , 2.00) |  |  |  |  |  |  |  |  |
|  | Stomach | 1.28 (0.72 , 2.08) |  |  |  |  |  |  |  |  |
| **Currie (2009) [3]** | All Cancer | 1.36 (1.19 , 1.54) | The Health Information Network (THIN) | Newly initiated metformin monotherapy for at least 6 prescriptions | at least 6 prescriptions of sulfonylurea | Monotherapy with metformin for a minimum specified period | Time updated (current/ever/cumulative) | Sulfonylurea | Yes | Mean 2.3 years |
|  | Breast | 0.98 (0.69 , 1.41) |  |  |  |  |  |  |  |  |
|  | Colorectal/Bowel | 1.80 (1.26 , 2.53) |  |  |  |  |  |  |  |  |
|  | Pancreas | 4.95 (2.74 , 8.96) |  |  |  |  |  |  |  |  |
|  | Prostate | 1.07 (0.76 , 1.49) |  |  |  |  |  |  |  |  |
| **Currie (2013) [47]** | All Cancer | 1.10 (1.00 , 1.20) | CPRD | Newly initiated metformin monotherapy for at least 180 days | sulfonylurea for at least 180 days | Monotherapy with metformin for a minimum specified period | Time updated (current/ever/cumulative) | Sulfonylurea | Yes | Mean 2.8 years |
| **Ferrara (2011) [48]** | Breast | 0.90 (0.80, 1.00) | Kaiser Perkanente Northern California Diabetes Registry (KPNC Diabetes registry) | 2 or more prescriptions of metformin in a 6 month period needed to define ever use. | no metformin | Any exposure but minimum time/number of prescriptions needed) | Time updated (current/ever/cumulative) | No metformin | No | Mean 3.6 years |
|  | Colorectal/Bowel | 1.00 (0.90 , 1.20) |  |  |  |  |  |  |  |  |
|  | Kidney | 1.30 (1.00 , 1.60) |  |  |  |  |  |  |  |  |
|  | Lung | 1.00 (0.80 , 1.10) |  |  |  |  |  |  |  |  |
|  | Melanoma | 0.80 (0.60 , 1.10) |  |  |  |  |  |  |  |  |
|  | Pancreas | 1.20 (1.00 , 1.50) |  |  |  |  |  |  |  |  |
|  | Prostate | 1.00 (0.90 , 1.10) |  |  |  |  |  |  |  |  |
| **Geraldine (2012) [49]** | All Cancer | 0.23 (0.09 , 0.60) | Intego database - Belgian primary care | Any exposure to metformin during the study period | diet only | Any Exposure | Single summary measure of exposure over entire follow up. | Diet | No | Mean 5 years |
| **Hense (2011) [50]** | All Cancer | 0.95 (0.90 , 1.01) | German D2C cohort | Metformin monotherapy at cohort entry | no metformin | Monotherapy with metformin | Fixed from start of follow up, with exposure occurring in a baseline period or follow up starting from first exposure | No metformin | No | Mean 3.5 years |
| **Home (2010) - Adopt [51]** | All Cancer | 0.92 (0.63 , 1.35) | ADOPT Clinical Trial | Randomisation to Metformin and Sulfonylurea | Randomisation to Rosiglitazone and Sulfonylurea | Randomisation to Metformin | Fixed from start of follow up, with exposure occurring in a baseline period or follow up starting from first exposure | Rosiglitazone | No | Mean 3.4 years |
| **Home (2010) -Record [51]** | All Cancer | 1.22 (0.86 , 1.74) | RECORD Clinical Trial | Randomisation to Metformin and Sulfonylurea | Randomisation to Rosiglitazone and Sulfonylurea | Randomisation to Metformin | Fixed from start of follow up, with exposure occurring in a baseline period or follow up starting from first exposure | Rosiglitazone | #N/A | Mean 5.5 years |
| **Hsieh (2012) [8]** | All Cancer | 0.56 (0.44 , 0.71) | Taiwan National Health Insurance Claims database | Metformin monotherapy for at least 1 year during study period | Sulfonylurea | Monotherapy for a minimum specified period | Single summary measure of exposure over entire follow up. | Sulfonylurea | No | Not reported |
|  | Breast | 0.57 (0.33 , 0.97) |  |  |  |  |  |  |  |  |
|  | Colorectal/Bowel | 0.54 (0.39 , 0.76) |  |  |  |  |  |  |  |  |
|  | Liver | 0.66 (0.49 , 0.91) |  |  |  |  |  |  |  |  |
|  | Lung | 0.64 (0.45 , 0.90) |  |  |  |  |  |  |  |  |
|  | Pancreas | 0.63 (0.28 , 1.42) |  |  |  |  |  |  |  |  |
|  | Prostate | 0.97 (0.60 , 1.55) |  |  |  |  |  |  |  |  |
|  | Stomach | 0.65 (0.39 , 1.08) |  |  |  |  |  |  |  |  |
| **Lai (2012) (HCC) [52]** | HCC/ICC | 0.49 (0.37 , 0.66) | Taiwan National Health Insurance Claims database | Any exposure to metformin during the study period | other oad | Any Exposure | Single summary measure of exposure over entire follow up. | Any other OAD | Not sure | Mean 5.28 years |
| **Lai (2012) (Lung) [53]** | Lung | 0.55 (0.37 , 0.82) | Taiwan National Health Insurance Claims database | Any exposure to metformin during the study period | other oad | Any Exposure | Single summary measure of exposure over entire follow up. | Any other OAD | Not sure | Mean 4.52 years |
| **Lee (2011) [54]** | Colorectal/Bowel | 0.36 (0.13 , 0.98) | Taiwan National Health Insurance Claims database | At least 2 prescriptions of metformin during study period to define ever exposure, but also measured overall duration to metformin and adjusted for this in the final model | other oad | Total Exposure (Number of prescriptions/time on metformin) | Time updated (current/ever/cumulative) | Any other OAD | Yes | Mean 3.8 years |
|  | Oesophagus | 0.44 (0.07 , 2.61) |  |  |  |  |  |  |  |  |
|  | Liver | 0.06 (0.02 , 0.16) |  |  |  |  |  |  |  |  |
|  | Pancreas | 0.15 (0.03 , 0.79) |  |  |  |  |  |  |  |  |
|  | Stomach | 1.41 (0.42 , 4.73) |  |  |  |  |  |  |  |  |
| **Lehman (2012) [55]** | Prostate | 1.68 (1.46 , 1.94) | Veteran Health Administration Health Care System | At least 180 days of metformin monotherapy | Sulfonylurea | Monotherapy for a minimum specified period | Fixed from start of follow up, with exposure occurring in a baseline period or follow up starting from first exposure | Sulfonylurea | No | Mean 5.2 years |
|  |  |  |  |  |  |  |  |  |  |  |
| **Libby (2009) [5]** | All Cancer | 0.63 (0.53 , 0.75) | DARTS,Tayside Medicines Monitoring Unit, Scottish Morbidity record | More than 1 prescription of metformin in study period | no metformin | Any Exposure | Fixed from start of follow up, with exposure occurring in a baseline period or follow up starting from first exposure | No metformin | No | Not reported |
|  | Breast | 0.60 (0.32 , 1.10) |  |  |  |  |  |  |  |  |
|  | Colorectal/Bowel | 0.60 (0.38 , 0.94) |  |  |  |  |  |  |  |  |
|  | Lung | 0.70 (0.43 , 1.15) |  |  |  |  |  |  |  |  |
| **Morden (2011) [56]** | All Cancer | 1.01 (0.94 , 1.08) | Medicare | Ever exposure to metformin in first 4 months after study enrolment | no metformin | Any Exposure | Fixed from start of follow up, with exposure occurring in a baseline period or follow up starting from first exposure | no met | No | Mean 1.93 years |
|  | Breast | 1.28 (1.05 , 1.57) |  |  |  |  |  |  |  |  |
|  | Colorectal/Bowel | 0.94 (0.72 , 1.22) |  |  |  |  |  |  |  |  |
|  | Pancreas | 1.25 (0.89 , 1.75) |  |  |  |  |  |  |  |  |
|  | Prostate | 0.97 (0.76 , 1.24) |  |  |  |  |  |  |  |  |
| **Morgan (2012) [64]** | All Cancer | 0.93 (0.76 , 1.16) | GPRD | Metformin & Sulfonylurea combination therapy as 2nd line treatment after metformin monotherapy | Sulfonylurea only as 2nd line treatment after metformin monotherapy | Metformin + Sulfonylurea combination therapy | Fixed from start of follow up, with exposure occurring in a baseline period or follow up starting from first exposure | Sulfonylurea | No | Mean 2.3 years |
| **Neumann (2011) [57]** | Bladder | 1.03 (0.93 , 1.14) | French national health insurance system - SBIIRAM (reimbursement database) and PMSI (hospital records) databases | At least 2 prescriptions of metformin over 6 consecutive months | other oad | Any exposure but minimum time/number of prescriptions needed) | Time updated (either no to yes or cumulative) | Any other OAD | No | Mean 3.1 years |
|  | Breast | 0.92 (0.88 , 0.97) |  |  |  |  |  |  |  |  |
|  | Colorectal/Bowel | 1.02 (0.98 , 1.07) |  |  |  |  |  |  |  |  |
|  | Kidney | 0.97 (0.89 , 1.05) |  |  |  |  |  |  |  |  |
|  | Lung | 0.88 (0.84 , 0.92) |  |  |  |  |  |  |  |  |
| **Oliviera (2008) [58]** | Bladder | 0.99 (0.70 , 1.39) | US insurance database | Ever use of metformin during follow up | no met | Any Exposure | Single summary measure of exposure over entire follow up. | No metformin | No | Mean 3.9 years |
|  | Colorectal/Bowel | 0.67 (0.52 , 0.85) |  |  |  |  |  |  |  |  |
|  | Liver | 0.73 (0.34 , 1.56) |  |  |  |  |  |  |  |  |
|  | Pancreas | 1.26 (0.80 , 1.99) |  |  |  |  |  |  |  |  |
| **Qiu (2013)** | All Cancer | 0.93 (0.86 , 1.02) | CPRD | at least 6 sequential prescriptions of metformin monotherapy | Sulfonylurea monotherapy | Monotherapy for a minimum specified period | Fixed from start of follow up, with exposure occurring in a baseline period or follow up starting from first exposure | Sulfonylurea | Not sure | Mean 3.8 years |
| **Redaniel (2012) [60]** | Breast | 1.04 (0.79 , 1.37) | CPRD | Monotherapy with metformin for at least 6 months | Sulfonylurea monotherapy | Monotherapy for a minimum specified period | Time updated (either no to yes or cumulative) | Sulfonylurea | Yes | Mean 4.96 years |
|  |  |  |  |  |  |  |  |  |  |  |
|  |  |  |  |  |  |  |  |  |  |  |
| **Ruiter (2012) [9]** | All Cancer | 0.90 (0.88 , 0.91) | PHARMO Record Linkage System (dispensing records from community pharmacies linked to hospital discharge records in Netherlands) | Monotherapy with metformin adjusting for cumulative use | Monotherapy with metformin adjusting for cumulative use | Total Exposure (Number of prescriptions/time on metformin) | Time updated (either no to yes or cumulative) | Sulfonylurea | Yes | Mean 3.5 years |
|  | Breast | 0.95 (0.91 , 0.98) |  |  |  |  |  |  |  |  |
|  | Colorectal/Bowel | 0.91 (0.88 , 0.94) |  |  |  |  |  |  |  |  |
|  | Oesophagus | 0.90 (0.82 , 0.97) |  |  |  |  |  |  |  |  |
|  | HCC/ICC | 0.67 (0.53 , 0.86) |  |  |  |  |  |  |  |  |
|  | Lung | 0.87 (0.84 , 0.91) |  |  |  |  |  |  |  |  |
|  | Pancreas | 0.73 (0.66 , 0.80) |  |  |  |  |  |  |  |  |
|  | Prostate | 0.92 (0.88 , 0.97) |  |  |  |  |  |  |  |  |
|  | Stomach | 0.83 (0.76 , 0.90) |  |  |  |  |  |  |  |  |
| **Tsilidis (2014) [61]** | All Cancer | 0.96 (0.89 , 1.04) | CPRD | New users of metformin monotherapy | New users of sulfonylurea monotherapy | Monotherapy for a minimum specified period | Fixed from start of follow up, with exposure occurring in a baseline period or follow up starting from first exposure | Sulfonylurea | Yes | Mean 5.1 years |
|  | Bladder | 0.88 (0.64 , 1.21) |  |  |  |  |  |  |  |  |
|  | Breast | 1.03 (0.82 , 1.31) |  |  |  |  |  |  |  |  |
|  | Colorectal/Bowel | 0.92 (0.76 , 1.13) |  |  |  |  |  |  |  |  |
|  | Oesophagus | 1.05 (0.71 , 1.56) |  |  |  |  |  |  |  |  |
|  | Liver | 0.85 (0.49 , 1.47) |  |  |  |  |  |  |  |  |
|  | Leukaemia | 0.86 (0.57 , 1.31) |  |  |  |  |  |  |  |  |
|  | Lung | 0.85 (0.68 , 1.07) |  |  |  |  |  |  |  |  |
|  | Melanoma | 1.26 (0.82 , 1.95) |  |  |  |  |  |  |  |  |
|  | Ovarian/Endometrial | 1.38 (0.74 , 2.57) |  |  |  |  |  |  |  |  |
|  | Pancreas | 0.70 (0.45 , 1.07) |  |  |  |  |  |  |  |  |
|  | Prostate | 1.02 (0.83 , 1.25) |  |  |  |  |  |  |  |  |
| **Van Staa (2012) [62]** | All Cancer | 0.82 (0.75 , 0.90) | CPRD | At least 60 months exposure to metformin | 0-6 months exposure to metformin | Total Exposure (Number of prescriptions/time on metformin) | Time updated (either no to yes or cumulative) | Less exposure | Yes | Mean 4.4 years |
|  | Breast | 0.82 (0.61 , 1.10) |  |  |  |  |  |  |  |  |
|  | Colorectal/Bowel | 0.96 (0.71 , 1.29) |  |  |  |  |  |  |  |  |
|  | Lung | 1.06 (0.80 , 1.41) |  |  |  |  |  |  |  |  |
|  | Pancreas | 0.11 (0.07 , 0.16) |  |  |  |  |  |  |  |  |
|  | Prostate | 0.69 (0.52 , 0.91) |  |  |  |  |  |  |  |  |
| **Yang (2011) [63]** | All Cancer | 0.45 (0.29 , 0.68) | Hong Kong Diabetes Registry | At least 1 prescription of metformin | no metformin | Any Exposure | Single summary measure of exposure over entire follow up. | No metformin | Not sure | Mean 5.5 years |
|  |  |  |  |  |  |  |  |  |  |  |

Supplementary table 4a: Full bias assessment for each case control study

| **Study** | **Case (Outcome) Definition** | **Control Selection** | **Exposure Definition** | **Treatment of HBA1c, BMI, Other meds** | **Other Baseline Adjustments** | **Missing data** |
| --- | --- | --- | --- | --- | --- | --- |
| **Azoulay (2011)** | unlikely | unlikely | unlikely | Medium - BMI, HbA1c and other meds are nearest measure to index date, with ever exposure measured before this. Likely that this will include measures of HbA1c and other medications on causal pathway between metformin treatment and cancer rather than adjust for confounding. | Medium - Covariates measured at index date not at cohort entry, which may not correctly adjust for differences at cohort entry. Smoking only binary which potentially lacks detail. No information on duration of diabetes prior to starting therapy. | Unlikely/unknown - percentage of missing covariates generally low (reported for BMI and smoking) unknown percentage of missing hba1c though. |
| **Becker (2013)** | unlikely | unknown - not clear whether controls were cancer free at matched index date or cancer free for entire follow up (the latter having potential to induce bias). | low - not incident diabetic/user cohort so potential for miss classification of use | Medium - No adjustment for HBA1C (as didn’t alter estimate when tested), adjustment for BMI and other OADs, but not clear when this BMI is measured relative to cohort entry or start of exposure. Likely to be on causal pathway rather than before treatment | Medium - Adjusted for diabetes duration but not clear how this was determined. Smoking measured with sufficient categorical detail. Matching did not take into account diabetes status, so in an analysis restricted to cases and controls with diabetes, it is not clear whether this matching was broken and/or whether matching factors were subsequently adjusted for. | unknown- Missing indicator method used for bmi and smoking but amount of missing data not reported (only reported for full cohort not diabetics only) |
| **Bodmer (2011)** | Low - although applied in a sensitivity analysis, when looking at diabetics only, potential latency of cancer not considered. | unknown - not clear whether controls were cancer free at matched index date or cancer free for entire follow up (the latter having potential to induce bias). | low - not incident diabetic/user cohort so potential for miss classification of use | Medium - adjustment for BMI but not clear when measured relative to cohort entry or start of exposure. Last recorded HbA1c before index date included, unlikely to correctly adjust for confounding by indication at time of exposure. | Medium - Adjusted for diabetes duration but not clear how this was determined. Smoking measured with sufficient categorical detail. Matching did not take into account diabetes status, so in an analysis restricted to cases and controls with diabetes, it is not clear whether this matching was broken and/or whether matching factors were subsequently adjusted for. | unknown - Missing indicator method used for bmi and smoking but amount of missing data not reported (only reported for full cohort not diabetics only) |
| **Bodmer (2010)** | Medium - no adjustment applied to allow for potential latency of cancer. Cancer must have occurred after use of OHA, however table 2 suggests patients on no treatment at all can be included. This means that cancers that should be attributed to no treatment (which contribute to the no metformin group) will be excluded disproportionately just because we know they later go on to use OHAs. | unlikely | medium - not matched on time in GPRD therefore potential that time for exposure ascertainment could be different in cases and controls - also, not incident users | Medium - adjustment for BMI but not clear when measured relative to cohort entry or start of exposure. Last recorded HbA1c before index date included, unlikely to correctly adjust for confounding by indication at time of exposure. | Low- Adjusted for age, gender, smoking and diabetes duration but not clear how the latter was determined - smoking measured with sufficient categorical detail. | unknown - Missing indicator method used for bmi and smoking but amount of missing data not reported (only reported for full cohort not diabetics only) |
| **Bodmer (2012) (pancreatic)** | Low - index date shifted back in primary analysis but not clear whether this shift was retained when analysis restricted to patients with diabetes only. | unknown - not clear whether controls were cancer free at matched index date or cancer free for entire follow up (the latter having potential to induce bias). | low - not incident diabetic/user cohort so potential for miss classification of use | Medium - No adjustment for HBA1C, adjustment for BMI and other OADs, but not clear when this BMI is measured relative to cohort entry or start of exposure. Likely to be on causal pathway rather than before treatment | Medium - Adjusted for diabetes duration but not clear how this was determined. Smoking measured with sufficient categorical detail. Matching did not take into account diabetes status, so in an analysis restricted to cases and controls with diabetes, it is not clear whether this matching was broken and/or whether matching factors were subsequently adjusted for. | unknown - Missing indicator method used for bmi and smoking but amount of missing data not reported (only reported for full cohort not diabetics only) |
| **Bodmer (2012) (Colorectal cancer)** | unlikely | unknown - not clear whether controls were cancer free at matched index date or cancer free for entire follow up (the latter having potential to induce bias). | low - not incident diabetic/user cohort so potential for miss classification of use | Medium - No adjustment for HBA1C (as didn’t alter estimate when tested), adjustment for BMI and other OADs, but not clear when this BMI is measured relative to cohort entry or start of exposure. Likely to be on causal pathway rather than before treatment | Low - Adjusted for age, gender, and smoking and diabetes duration but not clear how the latter was determined or where measured with respect to exposure - smoking measured with sufficient categorical detail. | Low - <15% missing for smoking and bmi with missing indicator method used. Sensitivity analysis performed to look at the impact of missingness on relative risk estimates but not clear what. |
| **Bodmer (2012) (Lung cancer)** | unlikely | unknown - not clear whether controls were cancer free at matched index date or cancer free for entire follow up (the latter having potential to induce bias). | low - not incident diabetic/user cohort so potential for miss classification of use | Medium - No adjustment for HBA1C, adjustment for BMI and other OADs, but not clear when this BMI is measured relative to cohort entry or start of exposure. Likely to be on causal pathway rather than before treatment | Medium - Adjusted for diabetes duration but not clear how this was determined. Smoking measured with sufficient categorical detail. Matching did not take into account diabetes status, so in an analysis restricted to cases and controls with diabetes, it is not clear whether this matching was broken and/or whether matching factors were subsequently adjusted for. | unknown- Missing indicator method used for bmi and smoking but amount of missing data not reported (only reported for full cohort not diabetics only) |
| **Bosco (2011)** | Low - No lag applied to allow for potential latency of cancer diagnosis. | unlikely | Medium - "none exposed" comparator group very mixed and mix of incident and prevalent users- potential for confounding by disease severity. Recent/Past metformin user potentially useful but no measure of overall length/strength of exposure after the 1 year minimum. Not matched on time in database so potential for differing time windows between cases and control. | Medium - no adjustment made for use of other drugs within those who were exposed to metformin. BMI proxy of clinical obesity measured as occurring at all during follow up, Blood pressure and HbA1c not measured. | Medium - smoking not adjusted for. Other adjustments made - "diabetes complications" as ever/never during follow up. All measured between diagnosis and index date so not clear how measurement relates to timing of exposure | unlikely |
| **Chaiteerakij (2013)** | Low - No lag applied to allow for potential latency of cancer diagnosis. | low - although hospital based, care taken to ensure controls representative of population from which cases were taken and sensitivity analysis performed to look at effect of calendar time differences in recruitment. Possible that within diabetic patients, those who are able to visit the hospital for regular clinics and therefore enrolled as controls are generally healthier and therefore more likely to be on metformin, as opposed to more intensive treatments in those who have to come to the clinic for cancer treatment. Since no demographics are displayed for met vs no met or within diabetics only, this cannot be assessed. | High - exposure only measured as ever/never in the 1 year preceding diagnosis. Highly unlikely that exposure at this time would affect the development of cancer so quickly, and strong potential to consider those who have been previously exposed to metformin but are no longer on it (potentially because of disease progression) to be classified as not exposed. | High - no adjustments made at all. | High- matched on age and gender but nothing else | unlikely |
| **Dabrowski (2013)** | Low - No lag applied to allow for potential latency of cancer diagnosis. | Medium - controls cancer free for entire follow up but then are matched to the index date of the case. | High - appears to be current treatment rather than ever treatment. Strong potential for reverse causality. No measure of duration/dosage of metformin | High - only adjusted for use of other drugs, however how this is measured is not clear. | High- matched on age and gender but nothing else | low - 6/59 (10%) excluded because of missing HbA1c |
| **Donadon (2010)** | Low - No lag applied to allow for potential latency of cancer diagnosis. | Medium- Diabetic controls admitted for things other than Diabetes or liver related diseases, large proportion were admitted for heart failure and hypertension - commonly caused by being overweight? Therefore possible that they are more likely to be on metformin and not a sulfonylurea (which can cause further weight gain) | High- measured at admission/cancer diagnosis. Strong potential for reverse causality. Only measured as yes/no with no idea of duration or dosage of treatment. | Medium - Adjustment for A1c , BMI at time of diagnosis/enrolment only so likely on causal pathway | Medium - smoking status not adjusted for. Age > < 65 only, | Low - alcohol is only covariate with missing data and this was only approx. 4% in the cases only. Some data on diabetes treatment missing in both control groups but percentage very low. |
| **Li (2009)** | Low - No lag applied to allow for potential latency of cancer diagnosis. | Low - Controls selected from healthy relatives/spouses/friends accompanying cases to hospital appointments. Not clear whether this group is representative of the population of patients with diabetes as a whole who are at risk of pancreatic cancer. | Medium - Obtained from patient recall, so some potential for recall bias - those with more advanced stage diabetes who stopped taking metformin a long time ago may forget that they used it. | Medium - Only BMI adjusted for, and ever use of insulin but not other OADs. BMI taken as a mean of self-reported BMI at three time points, so may not reflect differences in BMI at time of exposure or time of diagnosis. | Medium - Diabetes duration/severity measured but not adjusted for in model. Smoking adjusted for but with minimum detail (ever/never) and all measured by recall. | low - numbers in tables suggest there is missing data for some covariates and also in terms of exposure status but proportions don’t appear to be too large (not possible to work out exact missing amounts in patients with diabetes only) |
| **Evans (2005)** | Low - No lag applied to allow for potential latency of cancer diagnosis. | Medium - controls cancer free for entire follow up but then are matched to the index date of the case. | unlikely - with the exception of definition 1 (exposure in year before index date) which is unlikely to be a relevant risk period for a causal effect of metformin exposure on cancer risk | Medium - only BMI adjusted for, not clear at what time point it is measured. No info on other diabetes medications at all. | Low - all main baseline confounders included but not clear over what time interval they have been measured. | High - >25% missing for smoking, BMI and BP with missing category. Strong possibility for residual confounding. |
| **Hassan (2012)** | Low - No lag applied to allow for potential latency of cancer diagnosis. | Low - Controls selected from healthy relatives/spouses/friends accompanying cases to hospital appointments. Not clear whether this group is representative of the population of patients with diabetes as a whole who are at risk of pancreatic cancer. | Medium - assessed by patient recall and no time/level of exposure considered. Overall, duration of diabetes appears shorter in controls than cases therefore exposure time window may not be balanced between cases and controls | High - BMI, HBA1c and use of other drugs not considered at all. | Medium - smoking assessed from patient recall. Diabetes duration/severity not adjusted for | Unlikely - all information gathered by interview at one time point. Data requested unlikely to be unknown and therefore missing |
| **Margel (2013)** | Low - No lag applied to allow for potential latency of cancer diagnosis. | unlikely | Low - ever/never exposure potentially not representative of exposure that could actually affect risk of cancer, however cumulative duration definition better. | High - no adjustments made for HBA1c, BMI (unclear whether any of these co into the comorbidity index) | Unlikely - matched on diabetes duration which should account for baseline severity assuming measured well. Only missing adjustment is smoking status which may or may not be in the comorbidity index. | Unlikely - report relatively low percentage of missing covariate data and only for SES. |
| **Mazzone (2012)** | Low - No lag applied to allow for potential latency of cancer diagnosis. | Medium - controls cancer free for entire follow up but then are matched to the index date of the case. | Medium - duration only dichotomised as ever, or > 24 months. Matched on date of birth, but not time in database - if entire medical history of exposure not documented, this means there is potential for differing opportunities for exposure to be recorded between cases and controls. Also, not matched on length of diabetes diagnosis so quite possible that comparisons being made between patients at very different disease stages in terms of exposure. | Medium - BMI and A1c measured as mean through entire follow up. No adjustment for other medications such as sulfonylurea/insulin | Low - adjustments made but timing of measurements with respect to exposure is not clear. HbA1c is the only measure of "severity". Since not restricted to incident diabetes, adjustment for duration may also been useful. | unknown - Low levels of missing data for smoking, but not mentioned for other covariates |
| **Monami (2009)** | Low - No lag applied to allow for potential latency of cancer diagnosis. | Medium - controls cancer free for entire follow and recruited sequentially rather than at random. | Medium - mixture of medical records and self-reported exposure over the 10 years prior to cancer diagnosis/matched index date. Potential for recall bias. | Medium - matched on both BMI and HbA1c which were recorded at cohort entry. However, since patient recall of medication use before cohort entry used to inform exposure, this may not correct for confounding by indication, but may remove part of the total effect of metformin use on cancer risk. Only adjusted for ever use of other OHAs, timing of such a measurement makes interpretation unclear. | Unlikely - adjusted for duration of diabetes, smoking status age, gender and alcohol through matching | unlikely |
| **Monami (2011)** | Low - No lag applied to allow for potential latency of cancer diagnosis. | unlikely | Medium - well defined but low detail as only ever/never exposure to metformin. Only assessed exposure after insulin use so strong potential for misclassification of exposure to metformin. Some patient recall included. | Medium - bmi adjusted for by matching at insulin initiation. This does not necessarily precede metformin exposure since incident metformin use not established. No adjustment for HbA1c, and no adjustment for other oral OADS. Use of metformin or other OADs before insulin initiation was not considered. | Low- age and gender matched. Starting with incident insulin users will somewhat balance diabetes severity however CCS as a measure of severity of disease may not correctly adjust for differences between those who started metformin and not. | unlikely |
| **Smiechowski (2013)** | unlikely | unlikely | unlikely | Unlikely - cohort entry adjustments made for all TDCs and other medication entered as binary ever/never separately. Timing of measurement can be from 1 year before cohort entry up to 1 year before index date, therefore mixing baseline adjustment with adjustments that could potentially be on the causal pathway. A sensitivity analysis does address this and results are very similar. | Unlikely - all main baseline confounders included. Some potential to have adjusted for levels on causal pathway due to timing but sensitivity analysis performed to assess the impact of this. | unknown: approx. 18% missing HbA1c data for both cases and controls but not clear what ,if any, method used to deal with this |
| **Wang (2013)** | Low - No lag applied to allow for potential latency of cancer diagnosis. | Medium - controls cancer free until 2010 but then are matched to the index date of the case. | Low: Yearly median dose used in a secondary analysis but dichotomised only. Unknown how metformin use is actually established. | High: No adjustment for any potential TDC's | High - Only adjusted for age, gender and occupation - no adjustment for disease severity or smoking | Unlikely - not reported, but as an insurance database, age sex and occupation are unlikely to be missing. |
| **Chen (2013)** | Low - No lag applied to allow for potential latency of cancer diagnosis. | Low risk - those with previous liver surgery excluded, but was not applied to the cases. Not clear whether controls could have had a later HCC diagnosis | Low - observation period is only 5 years prior to index date, previous exposure not considered. | High- No adjustment for HbA1c or BMI. Use of other diabetic agents is included but measured during same window as total exposure so not clear whether adjustments are on causal pathway or not | Med- Age and gender matched, diabetes duration and severity (measured by number of visits) measured at index date. Severity in terms of number of visits could be affected by metformin use. Smoking not adjusted for. | unlikely - covariates used are yes no based on presence/absence of conditions, therefore not missing |
| **Donadon (2010) - 2** | Low - No lag applied to allow for potential latency of cancer diagnosis. | Medium- Diabetic controls admitted for things other than Diabetes or liver related diseases, large proportion were admitted for heart failure and hypertension - commonly caused by being overweight? Therefore possible that they are more likely to be on metformin and not a sulfonylurea (which can cause further weight gain) | High- measured at admission/cancer diagnosis. Strong potential for reverse causality. Only measured as yes/no with no idea of duration or dosage of treatment. | Medium - Adjustment for BMI at time of diagnosis/enrolment only. No adjustment for HbA1c and not clear how additional diabetes medications handled. | Low - smoking status not adjusted for, not entirely clear how diabetes duration was calculated. | unknown - no information provided |

Supplementary table 5b: Full bias assessment for cohort study

| **Study** | **Outcome Definition** | **Exposure Definition** | **Treatment of HBA1c, BMI, Other meds** | **Other Baseline Adjustments** | **Missing Data** | **Immortal Time** | **Censoring** |
| --- | --- | --- | --- | --- | --- | --- | --- |
| **Currie (2009)** | Low - No adjustment to allow for potential latency of cancer diagnosis. | High - future information (remaining on medication for 180 days if intensified or 6 prescriptions if initial therapy) used to define exposure at baseline and at any subsequent treatment change. Will result in exclusion of some individuals who do not continue on medication, either because they do not survive or because they are intolerant, high risk of inducing a selection bias. | Med - None of the key TDCs adjusted for in final model due to lack of statistical significance but timing of measures questionable. Once in cohort 3 and 4, all have previous use of an OHA, but duration of previous exposure and type of drug unknown. | Medium - No adjustment for diabetes severity at baseline, no distinction between current/ex-smoker. | Unknown: no information on level of missing data for any covariates | Low: minimum exposure of 6 consecutive prescriptions to be included in the study however follow up starts at date of first prescription, therefore immortal time exists in the study, however this immortal time occurs for every medication and comparisons only made between medication groups. Unclear whether the immortal time is largely different between sulfonylurea and metformin (depends on prescription length) and so whether it will heavily bias the estimate. | High: censoring occurs at treatment change and if next (intensified) treatment doesn’t last for 6 months they will be excluded from that cohort. Possible that reason for treatment not being continued related to outcome (e.g., they might already have cancer) |
| **Currie (2013)** | Low - No adjustment to allow for potential latency of cancer diagnosis. | High - future information (remaining on medication for 180 days) used to define exposure at baseline and at any subsequent treatment change. Will result in exclusion of some individuals who do not continue on medication, either because they do not survive or because they are intolerant, high risk of inducing a selection bias. | Low: Baseline adjustment made for HbA1c and BMI but does not consider previous use of OHAs which is necessary when time updating exposure between different groups. | Low: comprehensive list of potential baseline confounders but some with low detail - e.g. Smoking is only ever/never | unknown: multiple imputation used but no details given | Low: minimum exposure of 180 days but follow up starts from day 1. All cohort members must be on 1 drug so immortal follow up periods for everybody. | High: censoring occurs at treatment change and if next treatment doesn’t last for 180 days months they will be excluded from that cohort. Possible that reason for treatment not being continued related to outcome (e.g., they might already have cancer) |
| **Geraldine (2012)** | Low - No adjustment to allow for potential latency of cancer diagnosis. | High - ever metformin use (so binary only) exposure allocated at baseline as fixed but is based on entire follow up period. No minimum period needed for metformin, single prescription will suffice, so further potential to classify people as exposed even if they do not continue with medication after first prescription. | Low- adjusted for at baseline (with the exception of combined/other diabetes medication) - weight and HBA1c entered as continuous | Low- measured at baseline and with good detail, but missing adjustment for diabetes duration which is important here due to combining prevalent and incident cases. | Unknown - levels of missing data not reported | High -exposure definition means all follow up time before exposure starts still apportioned as "exposed" time | Unknown - number lost to follow up/death not recorded |
| **Home (2010)** | Unlikely - no latency period but treatment randomised therefore decision to treat not affected by potential pre-existing cancer. Some potential for including cancers unrelated to any treatment, however this would not be differential between treatment groups. | unlikely | unlikely- randomised at baseline so able to estimate a total effect of initial treatment allocation (but this does not answer the question of the causal effect as didn’t have 100% adherence) | Unlikely as treatment randomised | Unlikely | Unlikely | Low- RECORD <3% loss to follow up for unknown reasons. RECORD 526 subjects (out of over 4000) failed to adhere to monotherapy and were censored at this point which may have caused bias but the %age is quite low ADORT study - 21% non-adherence on metformin and 15% on rosiglitazone. Potential for a dilution of an effect but not likely to be huge? |
| **Hsieh (2012)** | Low - cancer in first year of follow up excluded to ensure cancer developed during study period, but first exposure could occur after this 1 year period. | High - Measured as monotherapy for at least 1 year on one of three treatments (for entire follow up). Excludes a large number of patients who will be on combination therapy at some point in their follow up - drop in n from 61,777 to 10,189 for the analysis suggests they have excluded anyone who has ever been on more than 1 drug or changed treatment. This choice of comparisons likely to be an a-typical group of patients - particularly those using insulin only. Cancer cases will have less follow up time to be exposed as if no cancer case the subject is followed up all the way until end of study period and logistic regression used. | High - not adjusted for at all. | High- adjusted for age and gender only. | Unlikely - not likely to have data on age and gender missing. | unlikely | Unknown - end of follow up classed as cancer diagnosis or 2008 (end of study). Not clear whether information on death / other censoring reasons available, but appear that the assumption made is if no cancer diagnosis then they are assumed alive and cancer free at end of study. |
| **Lai (2012) (HCC)** | Low - No adjustment to allow for potential latency of cancer diagnosis. | High - ever vs never exposure to metformin only, based on entire follow up period but assigned at baseline | Medium - No adjustment for use of other medications, no adjustment for HBA1c, though some baseline comorbidities adjusted for as yes/no presence (including obesity which could be a surrogate for BMI and partially adjust for severity of disease) | Medium - adjustment for age, gender but no smoking status and no other measure of disease severity. Due to how exposure defined, other adjustments (except age and gender) may not appropriately adjust for differences at time of treatment initiation. Insurance claims database so unhealthy behaviours likely to be unreported. | Unlikely - baseline covariate adjustments are yes/no for presence, where absence of information assumes no. | High -exposure definition means all follow up time before exposure starts still apportioned as "exposed" time | Unknown - number lost to follow up/death not recorded |
| **Lai (2012) (LUNG)** | Low - No adjustment to allow for potential latency of cancer diagnosis. | High - ever vs never exposure to metformin only, based on entire follow up period but assigned at baseline | Medium - No adjustment for use of other medications, no adjustment for HBA1c, though some baseline comorbidities adjusted for as yes/no presence (including obesity which could be a surrogate for BMI and partially adjust for severity of disease) | Medium - adjustment for age, gender, tobacco use and other comorbidity in form of propensity score, but no other measure of disease severity. Due to how exposure defined, other adjustments (except age and gender) may not appropriately adjust for differences at time of treatment initiation. Insurance claims database so unhealthy behaviours likely to be unreported. | Unlikely - baseline covariate adjustments are yes/no for presence, where absence of information assumes no. | High -exposure definition means all follow up time before exposure starts still apportioned as "exposed" time | Unknown - number lost to follow up/death not recorded |
| **Lee (2011)** | low - cancer only included if after 1 year of follow up from prescription, which will allow for latency of exposure effect/or of cancer diagnosis with regard to ever/never exposure to metformin, but may still be problematic when adjustments made for time updated duration of exposure. | High- follow up starts from first prescription of the drug for which they have been assigned based on knowledge of the entire follow up period. As such, the referent group is patients destined to never be on metformin, not those who may start on something else but end up on metformin. It is likely that these are people who have more severe disease and need to initiate straight onto stronger therapy, or those who have an absolute contraindication to metformin e.g. Renal disease, which may affect their underlying risk of certain cancers. | Med- adjusted for comorbidity score at baseline which may partially adjust for severity but not specifically BMI & HbA1c. Only adjustment for other medication is an ever/never exposure based on entire follow up - which by definition will always be "yes" in the referent group. | Medium - no adjustment for smoking status. Adjustment for "comorbidity" but this is general and not specific to disease severity. Age adjusted for with adequate detail. | Unlikely - baseline covariate adjustments are yes/no for presence, where absence of information assumes no. | Medium- Patients that start on an OAD other than metformin but move onto it later, have their survival time between first ever prescription and first metformin prescription censored. Therefore we systematically loose survival time which is cancer free (as they survive to go onto metformin) in that group, but cannot loose survival time in the metformin group. | low - only 2% loss to follow up (about the same in both metformin and referent group) |
| **Libby (2009)** | Low - No adjustment to allow for potential latency of cancer diagnosis. | High - When dosage included, it is maximum dose prescribed ever so potential for actual exposure levels for an individual to be miss classified. Choice of comparator group has potential to introduce bias due to selection process (non exposed selected from those who will never get metformin, not those who are not on metformin at matched index date) and allowing potential non exposed who would be excluded due to cancer before index date for one exposed subject to be matched to an exposed subject with a later metformin start date (therefore adding certain cancer events to the non exposed group). | Medium: using average of BMI and A1c throughout follow up likely to leave residual confounding by indication. Adjustment for use of other OADs at baseline will correctly adjust for baseline differences in previous medication use between those starting metformin and those not if the information on past use is accurate. | Unlikely - all key confounders measured. Aside from A1c and BMI (measured as means) they matched on year of diagnosis which as a diabetes registry may be more accurate than normal medical records | High: Unknown category included for smoking status, with 21 and 31% missing in exposed and unexposed respectively. | unlikely | Unknown: 34.8% of non-metformin users died from any cause as opposed to 14.9% of the metformin users, however it is not clear how many of these would have been censored due to death in the primary analysis. |
| **Qui (2013)** | unlikely- start of follow up is one year after first prescription, which ensures at least 1 year before outcome can occur (exposure defined as 6 consecutive prescriptions therefore it is possible that pre-existing cancer may have affected whether someone did or didn’t have stable therapy in that year, but overall bias from this is unlikely). | Medium - defined by "stable treatment initiation" only, so not necessarily incident users (although incident users only was included in a sensitivity analysis), and we also know that they will have six consecutive prescriptions. This means selection bias possible since many people to do not continue on medication long enough may be excluded. Additionally, if the 6 prescriptions are over a time of >1 year then future information will still have been used to define exposure at baseline. | Low - all assessed and measured in year prior to baseline (start of treatment) but not included in final model for outcome of all cancer. HbA1c and Weight were measured as highest over a year period which may not be correct for levels at time of treatment initiation which is why low and not unlikely. Also, univariate vs single adjustment tests done to decide inclusion, which may not accurately show if something is a confounder within a full multivariable model, so questionable whether excluding from final model was reasonable. | Low - Age and gender only, measures of disease severity at baseline (duration, number of prescriptions in previous year) and smoking status were considered but not included. Low rather than unlikely due to system of excluding confounders | Med - substantial missing data for A1c (bit less for BMI). The sensitivity analysis performed suggested that missing A1c and diabetes duration did not change the unadjusted estimates by more than 10% for all malignancies, yet no further discussion or methods used to assess to what extent this could affect overall conclusions. | Low - minimum 6 consecutive prescriptions to be included as exposed, but this is true for all included. If average time for 6 sulfonylurea prescriptions is systematically different to 6 consecutive metformin prescriptions (and if one of these is >1 year) then risk time could be disproportionately affected. | Unlikely - sensitivity analysis occurred with extra censoring at 6 months past change from initial treatment (or end of study if that occurred before) and this had little effect on the results. |
| **Redaniel 2012** | Unlikely - although not in primary analysis, exposure was re-examined including two different latency periods and results found to be similar, so care taken to insure bias not introduced by including outcomes in irrelevant risk periods. | Low - time varying exposure allows multiple cohort inclusion but within cohort no cumulative duration considered. | Medium -HbA1c adjusted for as an average, BMI adjusted for at baseline. No adjustment for use (even ever use) of other medication. | Med- Smoking not adjusted for. Age adjusted for with sufficient detail. HbA1c is only measure of disease severity and as mentioned in previous column this was done as an average which may not correctly adjust for confounding and may partially block causal pathway between exposure and outcome. | Unlikely. Multiple imputation used and well described. | Low - due to the condition for being "exposed" to a particular drug for > 6 months to be classed as exposed, there may be immortal time after entry to some categories that would not occur in others, however these categories are not included in the main analysis so only low risk. For example, if two patients start on sulfonylureas, and after 7 months one patient moves into the combination with metformin group, then we know that they must continue for 6 months more to have been classed as exposed to metformin as well as a sulfonylurea. | Unknown - loss to follow up due to death not reported. Sensitivity analysis of potential for ascertainment bias partly addresses issues with censoring directly at treatment change, as long latency periods are used (e.g.. 2/3 years which will account for more than just lag between actual development and diagnosis). |
| **Ruiter (2012)** | unlikely - different latency periods tested and no differences found | unlikely | High: HbA1c and BMI not considered as covariates at all. Use of other OADs not needed as an adjustment because they were an incident user group with ITT assumed and just cumulative use used as a time dependent covariate | Med: Number of hospitalisations used as a measure of comorbidity at baseline, but possible to have residual confounding from diabetes duration. Not able to adjust for smoking. I.e. Most of minimum set included (not smoking) but some with less than adequate detail. | Unknown - levels of missing data not reported | unlikely | Medium: censored at concomitant medication other than sulfs/metformin. Effect of exposure highly likely to be latent, so if there is a causal association, there is potential to miss cases by doing this. |
| **Tsilidis (2014)** | unlikely - used 12 month period after first exposure before follow up occurred, and changed to 6 months in sensitivity analysis | unlikely | Unlikely: No adjustment for baseline HbA1c in final model as included and found to not make much difference. Since incident group assuming ITT, this is reasonable to still estimate total effect of metformin vs sulf on cancer incidence. BMI adjusted for at baseline. Other medications not adjusted for but we assume ITT and then censor at treatment change from monotherapy. Baseline here is 1 year prior to index date, and HbA1c was a time weighted average so closest to time of initiation gets most weight therefore likely to be representative of A1c levels at time of treatment decision. | unlikely | Low - generally only small proportions of missing data (<15%) with the exception of alcohol consumption which is much higher (28%) with missing indicator method used (however alcohol not considered a key confounder so potential effect of this is probably small). No unadjusted effects reported, so unable to judge whether this method may leave residual confounding. | unlikely | Low - weighting applied to adjust for non-adherence but not for other reasons for censoring, but this only provides a valid adherence adjusted estimate if the model predicting adherence is correctly specified and there are no unmeasured causes of censoring and cancer. Since censoring applied on exact date of change of treatment, any change in treatment due to presence of pre-diagnosed cancer would not be accounted for. No information reported on numbers censored due to loss to follow up/death. |
| **Yang (2011)** | Low - No adjustment to allow for potential latency of cancer diagnosis. | High - ever/never metformin use based on entire follow up period but exposure allocated at baseline. | Low - all potential TDCs measured at cohort entry but since exposure is measured throughout follow up, and cohort entry is enrolment, the measures at this time point may not correctly adjust for differences at time of treatment initiation. | Unlikely - comprehensive list of baseline confounders that appear to be measured accurately at cohort entry (though slight uncertainty as to whether they are actually incident cases). | unknown - complete case analysis used but not clear how much missing data there was for covariates (only HDL cholesterol reported) | High -exposure definition means all follow up time before exposure starts still apportioned as "exposed" time | unknown - loss to follow up due to death not reported |
| **Buchs (2011)** | Low - No adjustment to allow for potential latency of cancer diagnosis. | High - Baseline period for exposure assessment from 2000 - 2002, with follow up exposure from 2003 onwards. However no requirement for included subjects to be newly diagnosed (just to have not had any insulin). Potential for bias induced by comparison of subjects at inherently different disease stages since period for exposure ascertainment somewhat arbitrary with respect to start of disease. Future information on dosage used to inform total dosage at baseline. | Medium - Only other medication adjusted for. | High- no adjustment for smoking or any measure of disease severity. Age dichotomised to < or > 65 only. | Unlikely - absence of measure would indicate a "no" rather than missing. | High - use of total number of prescriptions as a continuous predictor of exposure over all of follow up means those with more prescriptions could just be surviving to have them. | Medium - approx. 14% loss to follow up from health problems or leaving database. Therefore likely to have shorter exposure period and will not have a cancer diagnosis. |
| **Chiu (2013)** | Low - No adjustment to allow for potential latency of cancer diagnosis. | High - ever/never metformin use based on entire follow up period but exposure allocated at baseline. | High - not adjustment for HbA1c or BMI at all, not clear whether each medication comparison was modelled separately or whether all in one model , so possibly no adjustment for other medication usage either | Medium - no adjustment for smoking, there was adjustment for “selected comorbidities" but not clear when these were measured and there may still be residual confounding by specific diabetes severity. | Unlikely: absence of measure would indicate a "no" rather than missing. | High -exposure definition means all follow up time before exposure starts still apportioned as "exposed" time | Unknown - no information on loss to follow up because of death. |
| **Ferrara (2011)** | Low - initial 6 month or 12 month exclusion period does not exactly control for latency of cancer since exposure is time updated, and later changes in exposure could still be affected by pre-diagnosed cancer | Low - not incident users so may be some misclassification in terms of past exposure | Medium - All adjusted for baseline value with exception of BMI. As exposure is time dependent this may not correctly adjust for those who are treated later in time. | Low - most of the key variables adjusted for with the exception of diabetes duration which may be important since not an incident cohort. | Medium - approx. 20% missing HbA1c and approx. 18% ethnicity, with missing indicator method used | unlikely | Unknown - no information on loss to follow up because of death. |
| **Olivieria (2008)** | Low - No adjustment to allow for potential latency of cancer diagnosis. | High - only ever/never or current use vs current use of other drugs. Current/past definition is unclear and does not take into account latency effect - i.e. Even if someone stops taking metformin the effect on cancer will occur well beyond "past use" and as such not be included? Ever/Never exposure measured over entire follow up period as ever use so future information used to inform baseline exposure. | High - no adjustment at all | High - adjusted for age and sex only, nothing to assess disease severity | Unlikely - not likely to have data on age and gender missing. | High -exposure definition of ever/never means all follow up time before exposure starts still apportioned as "exposed" time | Unknown - number lost to follow up/death not recorded |
| **Hense (2011)** | Low - No adjustment to allow for potential latency of cancer diagnosis. | Medium - "medication at cohort entry" is current medication, so strong potential to miss people that have had prior exposure to medication - such people may be at differing stages of disease. | Low - adjusted for BMI (study entry) and other meds (study entry) but not HbA1c. | low - adjusted for all but smoking status | unlikely | unlikely | Medium - if no cancer record then assumed to have survived. This could potentially add extra survival time, particularly to those who are more severe (and have actually died of other causes) who would more likely be on multiple medications and therefore not on metformin monotherapy. |
| **Lehman (2012)** | Low - No adjustment to allow for potential latency of cancer diagnosis. | High - follow up starts from first metformin or sulfonylurea prescription, but definition requires 180 days of that prescription and no use of other drugs (TZD or insulin) and no more than 180 days of the comparator drug, for entire prescription period. High risk that this will induce selection bias by excluding people who do not fit this specific exposure pattern. Dose dependent analysis uses proportion of days at higher dose, which is relative not absolute so may not compare actual exposure levels correctly. | Medium- HbA1c measured as an average through follow up so not clear that this would adjust for differences at therapy initiation and could be adjusting for things on the causal pathway. No adjustment for BMI. | Low: diabetes duration only dichotomised, Smoking status appears to be defined as "on smoking cessation therapy" which may be less accurate. | Unknown: complete case analysis used, not clear what proportions of subjects were excluded due to missing data. | Low: minimum exposure of 180 days but fop starts from day 1. All cohort members must be on 1 drug so immortal follow up periods for everybody. | unknown - loss to follow up due to death not reported |
| **Liao (2012)** | Low - No adjustment to allow for potential latency of cancer diagnosis. | High - yes/no exposure to metformin during the follow up period so future information is being used to define exposure from baseline. | high: univariate analysis | high: univariate analysis | Unknown: no information on level of missing data for any covariates | Medium: immortal time between entry into cohort and time of medication included - everyone must be on an oad, so everyone will have time added but if time to medication differs between medication groups this will cause bias. | unknown - loss to follow up due to death/transfer from database not reported |
| **Morden (2011)** | Low - incident cases counted as those diagnosed after the 4 month exposure assessment window, but this does not ensure a minimum exposure of 4 months. Still potential for reverse causality. | Medium - all must have had an insulin prescription in the first 4 months of enrolment in the part D program. Meaning all have relatively advanced t2DM at entry. Metformin use only established within this window, and prior use not considered which may be important considering any effect would be expected to occur years after exposure. | Medium - HbA1c and BMI not considered at all but diabetes complications are used as an adjustment for severity which could be considered a proxy for HbA1c. | unlikely - key fixed confounders included and some proxy measures of severity | unknown - not clear how much missing data there was (though most covariates absence = no) | unlikely | unknown - loss to follow up not reported |
| **Neumann (2011)** | Low - No adjustment to allow for potential latency of cancer diagnosis. Since exposure is time updated, the 6 month "baseline" exposure does not guarantee that the exposure status could not be affected by undiagnosed cancer. | Low- no exposure information prior to 2006 and they are not incident diabetic cases so potential for misclassification. | High - HbA1c and BMI not considered at all | Low - Age gender and proxy for diabetes duration included, data on smoking not available. | Unlikely - no missing data reported for variables used in analysis | unlikely | unknown - loss to follow up not reported |
| **Van Staa (2012)** | Low - multiple analyses to assess patterns of risk, some of which may be affected by the fact that no latency period used, but others will not be affected by this. Some potential for main estimate used to be affected. | unlikely | Low - measured at baseline, all key included except HbA1c. | Unlikely - all key baseline confounders included. Nothing specific for diabetes severity but all patients are new users of OADs. Other comorbidities may partially account for diabetes severity. | Low - MIM used for BMI as categorical but presented as continuous mean (SD) so extent missing unknown. Probably quite low based on knowledge of BMI recording for diabetics in CPRD. Unknown smoking status low proportion missing. | unlikely | low/med: for between treatment comparisons censored directly at change time so cancer early in new exposure unlikely to be attributable to new exposure but results relating to long term use comparisons may not have this problem. |
| **Morgan (2012)** | Low - No adjustment to allow for potential latency of cancer diagnosis. | unlikely | Unlikely - all TDCs measured at cohort entry, so will adjust for differences between those who continue with metformin and those who switch. | unlikely - all key adjustments made at baseline | Unknown - only 0.3% missing data on smoking status. But missingness not reported for HbA1c and BMI - though have used CPRD so bound to be some! | Low: minimum exposure of 90 days but fop starts from day 1. All cohort members must be on 1 drug so immortal follow up periods for everybody. | Medium - Censored at change in therapy - potential to miss cancer cases occurring after this point. Loss to follow up for this reason and death etc. not reported so unclear how this may affect it but certainly there is a risk |
